# Revealing the molecular landscape of human placenta: a systematic review and meta-analysis of single-cell RNA sequencing studies

**DOI:** 10.1093/humupd/dmae006

**Published:** 2024-03-13

**Authors:** Emilie Derisoud, Hong Jiang, Allan Zhao, Pascale Chavatte-Palmer, Qiaolin Deng

**Affiliations:** Department of Physiology and Pharmacology, Karolinska Institutet, Solna, Stockholm, Sweden; Department of Physiology and Pharmacology, Karolinska Institutet, Solna, Stockholm, Sweden; Department of Physiology and Pharmacology, Karolinska Institutet, Solna, Stockholm, Sweden; INRAE, BREED, Université Paris-Saclay, UVSQ, Jouy-en-Josas, France; Ecole Nationale Vétérinaire d’Alfort, BREED, Maisons-Alfort, France; Department of Physiology and Pharmacology, Karolinska Institutet, Solna, Stockholm, Sweden; Center for Molecular Medicine, Karolinska University Hospital, Solna, Stockholm, Sweden

**Keywords:** marker genes, cell type annotation, single-nucleus RNA sequencing, single-cell RNA sequencing, trophoblasts

## Abstract

**BACKGROUND:**

With increasing significance of developmental programming effects associated with placental dysfunction, more investigations are devoted to improving the characterization and understanding of placental signatures in health and disease. The placenta is a transitory but dynamic organ adapting to the shifting demands of fetal development and available resources of the maternal supply throughout pregnancy. Trophoblasts (cytotrophoblasts, syncytiotrophoblasts, and extravillous trophoblasts) are placental-specific cell types responsible for the main placental exchanges and adaptations. Transcriptomic studies with single-cell resolution have led to advances in understanding the placenta’s role in health and disease. These studies, however, often show discrepancies in characterization of the different placental cell types.

**OBJECTIVE AND RATIONALE:**

We aim to review the knowledge regarding placental structure and function gained from the use of single-cell RNA sequencing (scRNAseq), followed by comparing cell-type-specific genes, highlighting their similarities and differences. Moreover, we intend to identify consensus marker genes for the various trophoblast cell types across studies. Finally, we will discuss the contributions and potential applications of scRNAseq in studying pregnancy-related diseases.

**SEARCH METHODS:**

We conducted a comprehensive systematic literature review to identify different cell types and their functions at the human maternal–fetal interface, focusing on all original scRNAseq studies on placentas published before March 2023 and published reviews (total of 28 studies identified) using PubMed search. Our approach involved curating cell types and subtypes that had previously been defined using scRNAseq and comparing the genes used as markers or identified as potential new markers. Next, we reanalyzed expression matrices from the six available scRNAseq raw datasets with cell annotations (four from first trimester and two at term), using Wilcoxon rank-sum tests to compare gene expression among studies and annotate trophoblast cell markers in both first trimester and term placentas. Furthermore, we integrated scRNAseq raw data available from 18 healthy first trimester and nine term placentas, and performed clustering and differential gene expression analysis. We further compared markers obtained with the analysis of annotated and raw datasets with the literature to obtain a common signature gene list for major placental cell types.

**OUTCOMES:**

Variations in the sampling site, gestational age, fetal sex, and subsequent sequencing and analysis methods were observed between the studies. Although their proportions varied, the three trophoblast types were consistently identified across all scRNAseq studies, unlike other non-trophoblast cell types. Notably, no marker genes were shared by all studies for any of the investigated cell types. Moreover, most of the newly defined markers in one study were not observed in other studies. These discrepancies were confirmed by our analysis on trophoblast cell types, where hundreds of potential marker genes were identified in each study but with little overlap across studies. From 35 461 and 23 378 cells of high quality in the first trimester and term placentas, respectively, we obtained major placental cell types, including perivascular cells that previously had not been identified in the first trimester. Importantly, our meta-analysis provides marker genes for major placental cell types based on our extensive curation.

**WIDER IMPLICATIONS:**

This review and meta-analysis emphasizes the need for establishing a consensus for annotating placental cell types from scRNAseq data. The marker genes identified here can be deployed for defining human placental cell types, thereby facilitating and improving the reproducibility of trophoblast cell annotation.

## Graphical abstract

**Graphical Abstract:**
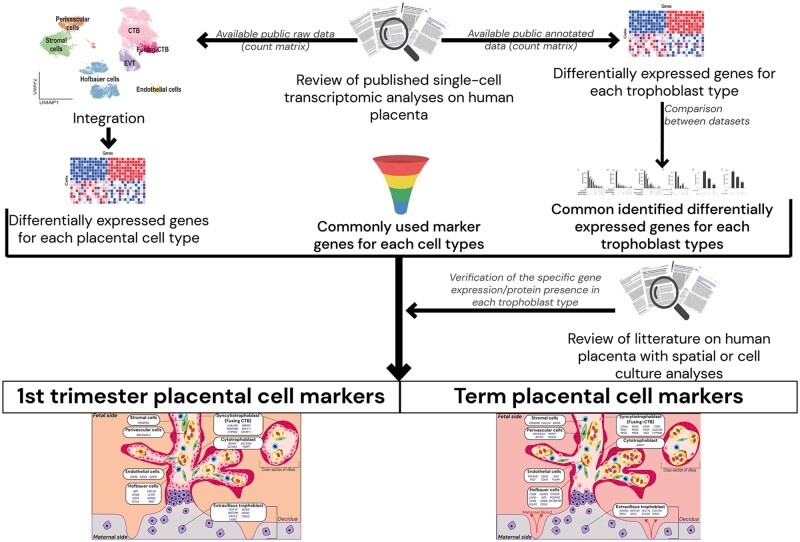
**Following the integration of existing single-cell transcriptomic data and a comprehensive literature review, we successfully identified marker genes crucial for defining distinct placental cell types. CTB: cytotrophoblast; EVT: extravillous trophoblast**.

## Introduction

Owing to its central and unique role at the interface of fetus and mother, placental dysfunction resulting from uterine environmental modifications could impact the short- and long-term health of the offspring. The association between altered fetal growth and the susceptibility to develop cardiovascular diseases in the adulthood was originally shown by Barker and Osmond (1986), Barker *et al.* (1989), and Barker (2007), becoming the foundation for the now well-known concept of Developmental Origins of Health and Diseases (DOHaD), also known as developmental programming. The placental growth, structure, and function both in animal models and humans have been shown to be strong predictors of cardiovascular and metabolic diseases in the adulthood (reviewed in Sferruzzi-Perri and Camm, 2016; Thornburg *et al.*, 2016), demonstrating the importance of the placenta for developmental programming.

Alteration of placental gene expression could be a key underlying cause of pregnancy pathologies and developmental programming effects. Single-cell RNA sequencing (scRNAseq) is a suitable technology to analyze placental cell type-specific changes with high resolution. However, it is not trivial to implement, with crucial steps being the recovery of high-quality single cells and proper annotation of each cell type. Despite easy accessibility of the placenta after delivery, the presence of elevated RNase levels poses a challenge to obtaining high-quality RNA for transcriptome analysis, which is highly dependent on appropriate tissue handling and freezing (Lanoix *et al.*, 2012; Huang *et al.*, 2013).

In this review, we have summarized the knowledge gained from published scRNAseq studies on the human maternal–fetal interface, as a reference for future studies. We have also presented the different cell types of fetal origin identified at the maternal–fetal interface by scRNAseq and compared previously defined markers for trophoblasts in first trimester and term placentas. Then, we integrated count matrices from the three available placental datasets of the first trimester and term placentas, respectively (i.e. 18 and 9 individuals). Through intersection with our meta-analysis of annotated markers from scRNAseq studies and relevant literature, we further curated a list of marker genes obtained from our integrated analysis of raw data. Finally, we discuss the contributions and potential applications of scRNAseq in studying pregnancy-related diseases.

## Origin and formation of the placenta

Placental development has previously been extensively reviewed (Huppertz, 2008; Boss *et al.*, 2018; Aplin *et al.*, 2020; Gauster *et al.*, 2022). Briefly, in humans, the attachment of the blastocyst to the uterine wall triggers differentiation of the polar trophectoderm into proliferative cytotrophoblasts (CTB) and multinucleated primary syncytium resulting from the fusion of CTB cells. This primitive syncytium invades the decidua to create fluid-filled spaces (lacunae) in which the syncytium continues to expand. This process forms the early structure of villi, the trabeculae.

During the second week post-fertilization, CTBs at the base of the primary syncytium project into the invaginations of the trabeculae, forming the primary villi. In the following days, mesenchymal cells invade the primary villi to induce secondary villi. Villi acquire their definitive structure (named tertiary villi) 3 weeks post-fertilization, composed of connective tissue, a vascular network, and macrophages (Hofbauer cells (HCs)) surrounded by trophoblasts. At the end of the first month of pregnancy, the basis of the placental structure is completed.

From the 8th week of gestation, a nearly uniform structure is observed among the villi, named mesenchymal villi owing to their high stromal proportion. They are the most primitive type of villi and are mainly responsible for the further growth of the villous tree with progression of pregnancy, although they also perform nutrient and gas exchange as well as endocrine activities (Castellucci and Kaufmann, 1982).

After the 8th week, the structure of the mesenchymal villi develops to form the immature intermediate villi. They are characterized by a very loose mesenchymal stroma together with HCs and developing vessels (Castellucci and Kaufmann, 1982). Immature intermediate villi are considered the starting point of all other villous types and the principal sites of nutrient and gas exchange during the first and second trimesters (Castellucci and Kaufmann, 1982).

After the 14th week of pregnancy, immature intermediate villi, located proximal to the chorionic plate, stop growing and instead develop into stem villi (Castellucci and Kaufmann, 1982). The function of stem villi is to support the structures of each villous tree and contain larger vessels leading the fetal blood into the ramifications (Demir *et al.*, 1997). One part of these villi, named anchoring villi, connects the villous tree to the decidua to supply the migratory CTBs that invade the decidua and blood vessels (reviewed in Aplin, 2010). Other villi that do not encounter the decidua are called floating villi.

From the second trimester, mature intermediate villi, with more capillaries and bigger fetal veins and arteries, are observed. They develop grape-like branches and give rise to terminal villi, characterized by an enlargement of their structure and a sinuosity of the fetal capillaries (Castellucci and Kaufmann, 1982). Capillary volume represents around 20% of the terminal villi (Burton *et al.*, 1996; Plitman Mayo *et al.*, 2016), allowing closer proximity of fetal blood to the maternal blood, and greatly facilitating exchanges. Expansion of the total volume and area of the placental villi is mainly related to growth of the terminal villi from the middle of the second trimester (Jackson *et al.*, 1992).

## Cell types in the human placenta

Although the decidua is an interesting tissue in the context of the maternal–fetal interface, most of the studies on the decidua concern immune cells. This review focuses on the cells of fetal origin; therefore, here we describe only the placental cells, namely trophoblast and non-trophoblast cells.

### Trophoblasts

Historically, three major types of trophoblast cells were identified in the placenta, i.e. CTBs, syncytiotrophoblasts (abbreviated STB or STBs when related to nuclei), and extravillous trophoblasts (EVTs) (Hamilton and Boyd, 1951).

#### Cytotrophoblast

CTBs have been considered proliferative as they are mitotic and express proliferative markers (Simpson *et al.*, 1992). CTBs form a continuous layer beneath the basement membrane of STB, producing a villous cytotrophoblast (vCTB) shell beneath the STB and cytotrophoblast cell columns (CCCs) at the distal end of the anchoring villi (Turco and Moffett, 2019). The vCTB shell progressively becomes discontinuous throughout the pregnancy (Kar *et al.*, 2007). At the end of pregnancy, vCTBs are sparse and only a thin STB layer separates maternal blood from the villous stroma and fetal endothelial cells in the terminal villi (Simpson *et al.*, 1992). However, the number of CTB nuclei increases throughout pregnancy, meaning CTBs remains proliferative toward term (Simpson *et al.*, 1992; Mayhew *et al.*, 1999).

#### Syncytiotrophoblast

STB is not proliferative (Bulmer *et al.*, 1988) but a highly polarized polynucleated epithelial layer (Gaunt and Ockleford, 1986). In the 1960s, vCTBs were hypothesized to be precursors for STB (Richart, 1961; Midgley *et al.*, 1963). This has been further revealed using trophoblast cell lines (Kliman *et al.*, 1986) and organoid cultures (Haider *et al.*, 2018; Turco *et al.*, 2018). Combining our current knowledge, STB is derived from asymmetrical cell division and differentiation of vCTBs followed by fusion with pre-existing syncytium (Knöfler *et al.*, 2019). To date, it is still unclear if the STB layer is composed of several cells or only one giant cell (GC) that covers the whole placental surface. STB is continuously and densely covered by microvilli that increase the exchange surface (Teasdale and Jean-Jacques, 1985; Karimu and Burton, 1995). STB is directly in contact with maternal glandular secretions during the first trimester of gestation and with maternal blood later in pregnancy (Burton *et al.*, 2002). In term placenta, the villous surface area of the placenta covered by STB has been estimated to be around 21 m^2^, which was likely largely underestimated owing to the microvilli (Luckhardt *et al.*, 1996). STB is responsible for most of the maternal–fetal exchanges during the pregnancy, with transporters for molecules, such as amino acids and glucose, on both apical and basal membranes, while large numbers of growth factor and hormone receptors are present on the microvilli membrane (Robinson *et al.*, 2009). STB is also responsible for the key endocrine functions of the placenta (Turco and Moffett, 2019). Importantly, STB does not express any HLA, essential to evade the maternal immune system (Moffett and Loke, 2006; Tersigni *et al.*, 2020). STB in term placentae, however, allows maternal IgG transfer, ensuring the maternal–fetal transfer of immunity (Leach *et al.*, 1996; Simister *et al.*, 1996).

#### Extravillous trophoblasts

CCCs become EVTs through contact with the decidua in anchoring villi (Aplin *et al.*, 1998), migrating either through the decidual stroma to the maternal spiral arteries, where they are classified as interstitial EVTs (iEVTs), or down to the inside of spiral arteries becoming endovascular EVTs (eEVTs) (Pijnenborg *et al.*, 1980). While CCCs are still round, iEVTs are pleomorphic and fusiform, containing four non-cycling nuclei showing senescent characteristics (Velicky *et al.*, 2018). iEVTs move toward the spiral arteries to establish a bulge of surrounding cells, participating in spiral artery remodeling (Turco and Moffett, 2019). Until the end of the first trimester, eEVTs form a plug at the entrance of the spiral arteries, preventing blood entry into the intervillous space (Boyd and Hamilton, 1970; Burton *et al.*, 1999).

Furthermore, aggregation and fusion of iEVTs occurs in the decidua to form GCs, a cell type whose origin, being fetal or maternal, has been questioned (Jones and Aplin, 2021). GCs can be observed as single large trophoblasts containing one or more nuclei in a voluminous cytoplasm or as mononuclear EVT aggregates separated by narrow intercellular spaces (Al-Lamki *et al.*, 1999). In first trimester placenta, they are observed in the decidua, close to the trophoblast shell of anchoring villi, but in term placenta, GCs localize at the junctional zone between the decidua and the myometrium (Jones and Aplin, 2021). They could have a role to limit trophoblast invasion but also ensure adequate local hormonal production, such as human placental lactogen, to maintain a normal pregnancy (Al-Lamki *et al.*, 1999).

### Fetal non-trophoblast cells

In addition to trophoblast cells, other cell types, such as vessel endothelium, Hofbauer, and connective cells, are known to exist in the core of the placental villi.

#### Stromal cells

Stromal cells were recently shown to originate from the embryonic hypoblast, but whether cells from the epiblast contribute or not is still undetermined (Boss *et al.*, 2018). Before the beginning of the second trimester, undifferentiated stromal cells expressing vimentin (Kohnen *et al.*, 1996) represent the main stromal cell type, but they are nearly absent in later pregnancy stages. From the second month of pregnancy, fully developed stromal cells, named reticulum cells, become the main stromal type in the terminal villi (Kaufmann *et al.*, 1977).

In the stem villi (i.e. villi at the fetal base of the placenta), fibroblasts are the main type of stromal cells. They differ greatly from the reticulum cells as they are specialized in producing collagen fibers and have very few cellular projections (Kaufmann *et al.*, 1977). The morphology of these cells suggests that, in addition to their supportive function, they have a role in stromal biosynthesis. They also can develop myofilaments, characteristic of myofibroblasts, with a structure that is intermediate between smooth muscle and fibroblast cells, with contractive abilities (Kohnen *et al.*, 1996).

#### Pericytes and vascular cells

Vasculogenesis and hematopoiesis occur in the villous core before the umbilical circulation is fully established, meaning that peri-vascular and vascular cells also originate from undifferentiated mesenchymal cells (Boss *et al.*, 2018). The lumen of the first vessel is observable from the 23rd day of pregnancy (Demir *et al.*, 1989). By the 32nd day of pregnancy, the vascular system connects to the fetus *via* the connective stalk, forerunner of the umbilical cord. The establishment of blood flow in the umbilical cord occurs by the end of the 5th week of gestation (Kaufmann *et al.*, 2004). Thereafter, during the second half of pregnancy, angiogenesis appears to be nearly absent (Demir *et al.*, 1989).

Simultaneously, stromal cells also differentiate into pericytes that are fibroblast-like cells intimately associated with endothelial cells. Pericytes are known to be responsive to changes in the local environment, with their presence/absence determining plasticity and sensitivity to soluble growth-factors derived from capillaries (Zhang *et al.*, 2002). The proportion of vessels associated with pericytes increases from 37% in the first trimester to 63% at term. Furthermore, vessels associated with pericytes present with a larger median diameter at term (Zhang *et al.*, 2002).

In the third month of pregnancy, a thin media- and adventitia-like structure develops in the stroma around the villous arteries and veins with a diameter larger than 100 µm, forming a fibrosis-like stromal core (Kaufmann *et al.*, 2004). The smooth muscle cells in the tunica media-like structure of the core have contractile abilities (Krantz and Parker, 1963). Moreover, the smooth muscle cells derived from placenta have a similar phenotype to aortic smooth muscle cells (Leik *et al.*, 2004). Collectively, this makes it unlikely that the feto–placental circulation is non-reactive to changes, as it is implied that the existing smooth muscle can control blood flow in the conceptus. However, as there are no shunts in the placental circulation, this function must be tightly controlled in order to prevent hypoperfusion in the placenta (Sebire and Talbert, 2002).

#### Hofbauer cells

Concomitant with the capillary development, as the only immune cells of fetal origin (Kim *et al.*, 2008) described in the placenta, HCs are specific macrophages and can also differentiate into stromal cells (Dempsey, 1972; Burton, 1987). HCs are present in placenta at all stages of pregnancy from as early as 18 days post-fertilization (Castellucci *et al.*, 1987; Thomas *et al.*, 2023). HCs are first produced by the placenta through primitive hematopoiesis but then further waves of colonization from the yolk sac and fetus occur as gestation progresses (Boyd and Hamilton, 1970; Castellucci *et al.*, 1987; Thomas *et al.*, 2023). Similar to other macrophages, HCs exhibit plasticity and pleomorphism depending on their environment (Reyes and Golos, 2018). They are mainly located adjacent to both endothelial and trophoblast cells (Castellucci *et al.*, 1980) but seem to be mobile around the villous stroma (Ingman *et al.*, 2010). During the first half of the pregnancy, HCs often undergo mitosis (Castellucci *et al.*, 1987). As macrophages, their primary role is likely protection of the fetus; however, only 7–8% seem able to phagocyte exogenous antibodies (Frauli and Ludwig, 1987) and it is unclear if HC control infection or clear placental damage (Reyes and Golos, 2018). HCs may also mediate placental morphogenesis and homeostasis as they can stimulate trophoblast growth, differentiation, and the secretion of hCG as well as human placental lactogen (hPL) in coculture with human CTBs (Khan *et al.*, 2000). Moreover, the HC number is correlated to the vasculogenic structures (Seval *et al.*, 2007), suggesting their involvement in vasculogenesis and angiogenesis.

## Methods

An extensive literature search using PubMed was performed to find articles related to human placental single-cell (sc) or single-nucleus (sn) RNA sequencing. Studies before 31 March 2023 were included in this systematic review and meta-analysis. The detailed procedure for the meta-analysis is described in Supplementary File S1. Furthermore, we also performed an analysis of published reviews in this field to include more relevant articles. When searching data deposits, if a new article was identified, it was also included in the meta-analysis. A list of articles analyzed in this meta-analysis is available in Supplementary Table S1.

## Results of qualitative analysis of current data on single-cell RNAseq of human placentas: systematic review and meta-analysis

The initial research retrieved 667 articles (633 from PubMed and 34 from the reviews; Li *et al.*, 2020; Barrozo and Aagaard, 2021; Chen *et al.* 2023; Supplementary Fig. S1). After removing duplicates, 421 articles were eligible for screening. A further three articles were added to the list as they also met the criteria. After the initial review using titles and abstracts, 281 articles were found irrelevant. Thereafter, 112 articles failed to fit the criteria (i.e. human tissue sampling, new original scRNAseq- or snRNAseq data, and fetal cells from placentas) and were excluded. Among the remaining articles, one analyzed only hematopoietic cells, and was hence excluded.

### scRNAseq of human placentas

The placenta has long been understudied despite its importance in many pregnancy-related complications and pathologies, and it is scarcely included in large-scale ‘omic’-analyses. For instance, the Genotype-Tissue Expression (GTEx) project, which performed an in-depth characterization of genetic associations, gene expression, and splicing in 49 tissues over 838 individuals, has not analyzed the placenta to date (The GTEx Consortium, 2020). Furthermore, while the first scRNAseq method was developed in 2009 (Tang *et al.*, 2009), the first scRNAseq analysis on human placenta was not published until 2017 (Pavličev *et al.*, 2017): this study profiled only 87 cells from two placentas collected after cesarean section from healthy pregnancies and used the C1 fluidigm platform with Smart-Seq2. To capture the multinucleated and giant STB, two cells were obtained through laser microdissection. However, it is not clear how the authors isolated STB and the number of collected nuclei. Interestingly, this limited number of cells still enabled the identification and comparison of gene expression in five clusters, including three distinct vCTB clusters, maternal dendritic cells, and EVTs in addition to STB. The most differentially expressed genes (DEGs) between cell types were, however, rRNAs, which newer library construction methods generally exclude before cDNA synthesis.

Later in 2017, another scRNAseq study using cesarean section-delivered placentas from two female and two male babies of both healthy and early pre-eclampsia pregnancies was performed (Tsang *et al.*, 2017). Using the 10× Genomics droplet-based method (referred to here as 10×), 20 518 cells were captured and annotated into 11 distinct major cellular subtypes from which maternal or fetal origin was determined either by single-nucleotide polymorphism analysis or Y-chromosome-linked gene transcription (Tsang *et al.*, 2017). With more cells captured compared to the first study, in-depth analyses revealed the developmental relations of the trophoblast cell types and their differentiation trajectories, in addition to the identification of 12 cell types in total. Since these two early studies, 26 more studies have used scRNAseq for analysis of the maternal–fetal interface (Table 1).

**Table 1.** Summary of the published placental single-cell or single-nucleus datasets of human maternal–fetal interface tissues.

	Reference	Sample information					Number of cells	Method	Data repository
		Precise GA	Tissue	Pathology	Delivery method	Number			
First trimester (<13 weeks of gestation)	(Suryawanshi *et al.*, 2018)	6–11	Placental villi	Healthy	Manual vacuum aspiration or dilation and curettage	5	14 341	Single cell, 10× Genomics and Drop-seq	BioProject ID: PRJNA492324
			Decidua				6754		
	(Liu *et al.*, 2018b)	8	Placental villi	Healthy	–	–	1471 (including first and second trimester placentas)	Single cell, FACS, SMART-seq2	GEO: GSE89497
	(Vento-Tormo *et al.*, 2018)	4–12	Placental villi	Healthy	Manual vacuum aspiration or dilation and curettage	5 (2 ♂ and 3 unknown)	18 547	Single cell, SMART-seq2 and 10× Genomics	ArrayExpress: E-MTAB6701 (10x Genomics) & E-MTAB-6678 (SMART-seq2)
			Decidua	Healthy		11 (5 ♀ and 4 ♂ & 2 unknown)	40 512		
	(Sun *et al.*, 2020)	10–13	Placental villi	Healthy	–	3 ♀ and 3 ♂	7245	Single cell, 10× Genomics	GEO: GSE131696
	(Han *et al.*, 2020)	10 and 13	Placenta	Healthy	Abortion	1 ♀ and 1 ♂	19 493	Single cell, Microcell-seq	GEO: GSE134355
	(Saha *et al.*, 2020)	6–8	Placental villi	Healthy	Abortion	2	–	Single cell, 10× Genomics	GEO: GSE145036
	(Du *et al.*, 2021)	6–9	Decidua	Healthy & RPL	Elective termination	6 RPL + 5 healthy	66 078	Single cell, 10× Genomics	BioProject ID: PRJNA673002
	(Shannon *et al.*, 2022)	5–12	Placental villi	Healthy	–	7 (4 ♀ and 3 ♂)	12 794	Single cell, 10× Genomics	GEO: GSE174481
			hTSC-derived trophoblast organoids			3 (2 ♀ and 1 ♂)	2628		
			EVT-differentiated hTSC-derived organoid			3	3726		
	(Zheng *et al.*, 2022)	5–12	Placental villi + decidua	Early miscarriage & healthy	Vacuum aspiration	20	218 899	Single cell, 10× Genomics	GEO: GSE174399
	(Liu *et al.*, 2022)	8	Placental villi	Healthy	Vacuum aspiration	2	–	Stereo-seq, STOmics Gene Expression	On author request
	(Pan *et al.*, 2022)	7–8	Maternal–fetal interface	Healthy & RPL	–	3 controls and 3 RPL	63 249	Single cell, BD Rhapsody™	None
	(Arutyunyan *et al.*, 2023)	4–13	Decidua	–	Elective termination (surgical and medicated)	8 (5 sc and 4 sn)	319 740 cells and 180 721 (+ 54 513 in multiome) nuclei (including peripheral blood and myometrium)	Single cell and single nuclei, 10× Genomics RNA and multiome + spatial transcriptomics, 10× Genomics Visium	ArrayExpress: E-MTAB-12421, E-MTAB-12595
			Placenta			4 (3 sc and 1 sn)			
Second trimester (13–26 weeks of gestation)	(Liu *et al.*, 2018b)	24	Placental villi	Healthy	–	–	1471 (including first and second trimester placentas)	Single cell, FACS, SMART-seq2	GEO: GSE89497
	(Pique-Regi *et al.*, 2020)	18	Decidua	Placenta accreta	–	2	A total of 13 190	Single cell, 10× Genomics	NIH dbGAP: phs001886.v2.p1
			Placental villi						
	(Cao *et al.*, 2020)	13–18	Placenta	–	–	6 ♀ and 5 ♂	29 876	Single nuclei, Sci-RNA-seq3	GEO: GSE156793
	(Marsh *et al.*, 2022)	18–24	Villous and smooth chorion	Healthy	Induced abortion	4	50 496	Single cell, 10× Genomics	GEO: GSE198373
Term (>26 weeks of gestation)	(Pavličev *et al.*, 2017)	39	Placenta	Healthy	Caesarian-section	2	87	Single cell, Fluidigm C1 and laser	GEO: GSE87726
	(Tsang *et al.*, 2017)	28–38	Placenta	Healthy and PE	Caesarian-section	2 ♀ and 2 ♂	20 518	Single cell, 10× Genomics	EBI: EGAS00001002449
	(Pique-Regi *et al.*, 2019)	33–40	Chorioamniotic membranes	With or without labor or preterm labour	Depending on the group	9 (3/group)	29 921	Single cell, 10× Genomics	NIH dbGAP: phs001886.v1.p1
			Placental villi				19 250		
			Basal plate				28 735		
	(Pique-Regi *et al.*, 2020)	36.9*	Basal plate (including chorionic villi and decidua)	–	Caesarian-section and normal delivery	2 × 2 datasets of 16 pooled decidua and placenta	13 305 nuclei	Single nuclei, 10× Genomics	NIH dbGAP: phs001886.v2.p1
	(Huang *et al.*, 2020)	39– 40	Decidua	Healthy	Cesarean without labor and vaginal	6 (3/delivery type)	29 231	Single cell, 10× Genomics	On author request
	(Lu-Culligan *et al.*, 2021)	38–40	Middle section and decidua	Healthy and SARS-CoV-2	Caesarian section and normal delivery	n = 3 healthy n = 2 SARS-CoV-2	83 378 (44 140 placenta and 39 238 decidua)	Single cell, 10× Genomics	GEO: GSE171381
	(Zhang *et al.*, 2021a)	30–36	Placenta	Healthy and PE	Caesarian section	3 controls and 3 PE	11 518	Single cell, GEXSCOPE	On author request
	(Yang *et al.*, 2021)	38–40	Placenta	Healthy and GDM	Caesarian section	2 controls and 2 GDM	27 220	Single cell, 10× Genomics	GEO: GSE173193
	(Zhou *et al.*, 2022)	32–40		PE		2	16 379		
	(Zhang *et al.*, 2022)	37–40		Old women		2	14 978		
	(Chen *et al.*, 2022)	28–30	Placenta	Healthy and SARS-CoV-2	Cesarean section	n = 2 healthy (2 ♂) n = 1 SARS-CoV-2 (♀)	17 481	Single cell, 10× Genomics	GSA: HRA000830
	(Garcia-Flores *et al.*, 2022)	38–40	Chorionic membranes, placental villi, basal plate	Healthy and SARS-CoV-2	Caesarian section and normal delivery	n = 11 healthy n = 12 SARS-CoV-2	–	Single cell, 10× Genomics	NIH dbGAP: phs001886.v3.p1
	(Wang *et al.*, 2022)	38–40	Fetal-side section	Healthy	Normal delivery	8	3490	Single cell, 10× Genomics	CSNA: CNP0000878
			Middle section				5304		
			Maternal-side section				2644		
	(Campbell *et al.*, 2023)	Term	Placental villi	Healthy	Cesarean	2	9244	Single cell, 10× Genomics	GEO: GSE182381

EVT: extravillous trophoblast; GA: gestational age; GDM: gestational diabetes mellitus; hTSC: human trophoblast stem cell; PE: pre-eclampsia; RPL: recurrent pregnancy loss; SARS-CoV-2: severe acute respiratory syndrome coronavirus 2; sc: single-cell; sn: single-nucleus; – indicates that the value is missing.

Among these studies, Vento-Tormo *et al.* (2018) performed the most extensive first-trimester single-cell profiling, analyzing 70 000 single cells with matched maternal blood and decidual cells. Pique-Regi *et al.* (2019) performed the most extensive scRNAseq study on term placentas, analyzing 79 906 cells of both maternal and fetal origin from nine women at different weeks of gestation with and without labor before delivery. Very recently, spatial transcriptomic analyses on placenta and decidua in the first trimester of pregnancy were performed (Liu *et al.*, 2022; Arutyunyan *et al.*, 2023).

Four reviews have been published on this topic (Li *et al.*, 2020; Xiao *et al.*, 2020; Barrozo and Aagaard, 2021; Chen *et al.*, 2023). The first one mainly explains the principles of scRNAseq and how it has been adapted in studies related to placentas (Li *et al.*, 2020). The second one mainly focuses on trophoblast cell differentiation (Xiao *et al.*, 2020). The third one presents how scRNAseq has been used in placental analysis to date and highlights the immunity and inflammation mechanisms previously observed in scRNAseq that can be linked to host–microbial interactions in the placenta (Barrozo and Aagaard, 2021). The authors also compiled a list of marker genes for more than 90 placental cell types and subtypes plainly based on the afore-mentioned studies without further analysis. The latest study mainly explains the principles of scRNAseq and emphasizes the applications in decidua (Chen *et al.*, 2023).

Recently, a meta-analysis on 11 bulk RNA sequencing and two microarray datasets on human trophoblast cultures and placenta collections demonstrated that *in vitro* production of trophoblast is dependent on the method used and that the expression of well-known trophoblast markers, such as *KRT7* or *CDX2*, is not sufficient to define trophoblasts. They also highlighted the need for consensus on markers and functional assays to assess *in vitro* trophoblast identity (Cox and Naismith, 2022).

Unlike the previous reviews, here we have systematically analyzed all the studies and highlighted the consensus and differences regarding sampling strategies, data analysis methods, and reported signature genes, aiming to provide a base of knowledge for future placental studies.

### Sample collection

#### Gestational age and delivery method

The period of collection of samples is highly variable across the published studies. Altogether, 14 studies analyzed third trimester placentas (>26 weeks of gestation). Term placentas are normally the easiest to obtain as they are considered biological waste at delivery in most countries and provide collective information about the entire pregnancy, but the delivery method may impact the transcriptome. Indeed, Pique-Regi *et al.* (2019) showed that macrophages and EVTs are particularly affected by the presence of labor or not at childbirth.

Term placenta can also be considered as too late a time point to study since several gestational disorders result from poor placental establishment and function earlier in pregnancy. Thus, first trimester (<13 weeks of gestation) analysis could be more informative and 12 scRNAseq studies used this gestational age (GA) (Liu *et al.*, 2018b; Suryawanshi *et al.*, 2018; Vento-Tormo *et al.*, 2018; Han *et al.*, 2020; Saha *et al.*, 2020; Sun *et al.*, 2020; Du *et al.*, 2021; Liu *et al.*, 2022; Pan *et al.*, 2022; Shannon *et al.*, 2022; Zheng *et al.*, 2022; Arutyunyan *et al.*, 2023). Decidua collected along with first trimester placentas was also investigated in six studies (Suryawanshi *et al.*, 2018; Vento-Tormo *et al.*, 2018; Du *et al.*, 2021; Pan *et al.*, 2022; Zheng *et al.*, 2022; Arutyunyan *et al.*, 2023). However, these types of samples are only available through abortions or miscarriages. Notably, recent debates around the legality of abortion in certain countries may lead to limitations on access to samples. Additionally, induced abortions could potentially impact the condition of the placenta and the cells within it because of exposure to drugs and other factors.

So far, only four studies (Liu *et al.*, 2018b; Cao *et al.*, 2020; Pique-Regi *et al.*, 2020; Marsh *et al.*, 2022) have analyzed second trimester placentas (13–26 weeks of gestation). These samples are particularly difficult to obtain as they either arise from late induced abortions, which are not authorized in several countries except under certain conditions, or from late miscarriages, for which samples can be difficult to obtain owing to the circumstances.

#### Sex of fetus

Only seven scRNAseq studies provided information of the fetal sex (Tsang *et al.*, 2017; Vento-Tormo *et al.*, 2018; Cao *et al.*, 2020; Han *et al.*, 2020; Sun *et al.*, 2020; Chen *et al.*, 2022; Shannon *et al.*, 2022). However, increasing evidence has shown that fetal sex is associated with differences in placental structure, function, and response to pathologies. An investigation of 88 649 deliveries over 30 years in Scotland highlighted that the placental weight and efficiency (defined as the ratio of fetal/placenta weight) are higher in male offspring (Wallace *et al.*, 2013). Moreover, using microarray and bulk RNA-sequencing analysis, differences in the placental transcriptome were observed according to fetal sex (Sood *et al.*, 2006; Gonzalez *et al.*, 2018). Furthermore, in male, but not female, fetuses from obese women, the placental unsaturated fatty acid uptake was inadequate in relation to the fetal growth (Brass *et al.*, 2013). Higher expression of inflammatory, hypoxic, and apoptotic molecules as well as reduced expression of pro-angiogenic markers were observed in severe pre-eclamptic placentas from male compared to female fetuses (Muralimanoharan *et al.*, 2013). Interestingly, the only scRNAseq study focusing on fetal sex in the first trimester placentas identified specific markers in certain cell types, such as *MUC15*, *NOTUM*, or *MAGEA4* in trophoblasts cells, which were affected differentially by the fetal sex, demonstrating the importance of the sex for the placental cell type annotation (Sun *et al.*, 2020). Furthermore, several genes related to the X or the Y chromosomes (i.e. *MAGEA4*, *TMSB4X*, *XIST*, *DDX3Y*, *EIF1AY*, or *RPS4Y1*) were differentially expressed in different cell types according to the fetal sex as expected (Sun *et al.*, 2020). These results highlight the importance of considering the sex as a parameter in maternal–fetal interface studies.

#### Sampling sites

One of the key considerations for scRNAseq studies on the maternal–fetal interface is the sampling site as the term placenta typically has a diameter of around 20 cm and a thickness of several centimeters. Most studies highlighted in our review do not report the site of the sampling in relation to the umbilical cord. Some scRNAseq studies analyze specific layers (i.e. decidua or placenta divided into fetal, internal, and maternal parts) but without further details. These analyses demonstrated that the proportion of some cell types varied according to vertical sampling site (Pique-Regi *et al.*, 2019; Wang *et al.*, 2022) and one of them identified different subtypes of CTBs and STBs between the compartments (Wang *et al.*, 2022). Using proteomics, 374 proteins were found to be differentially abundant between the maternal, internal, and fetal part of the placenta (Manna *et al.*, 2023). However, a recent study using bulk RNA sequencing did not observe any spatial variation, in either vertical or horizontal planes, in overall gene expression (Suryawanshi *et al.*, 2022). Lack of information about sampling sites may therefore lead to difficulty in comparisons between studies, but further investigation is needed to confirm this.

### Data collection and quality

**Figure 1.**
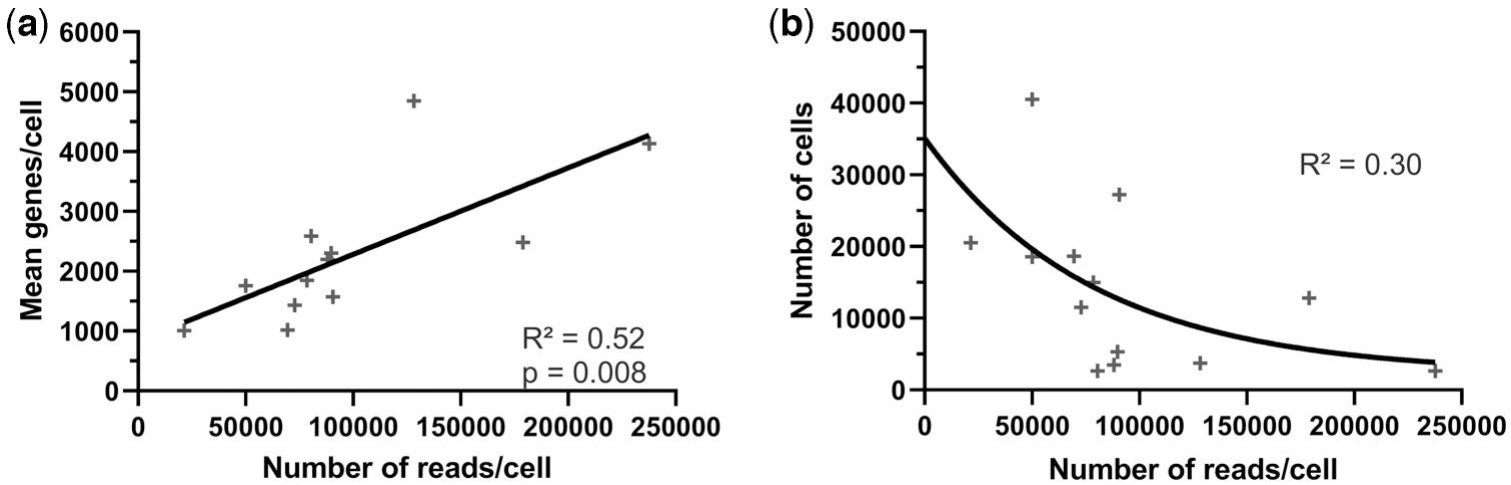
**Regression between the number of reads/cell and the mean genes/cell or the total number of cells.** Data from single-cell RNA sequencings of human placentas using 10×. (**a**) Reads/cell compared to the mean genes/cell; (**b**) reads/cell compared to the total number of cells; *R*^2^ calculated using linear (a) or exponential one-phase decay (b) least squares regression.

So far, most of the studies used the 10× method, and only eight studies used other methods (Drop-Seq, Smart-seq2, microcell-seq, Sci-RNA-seq3, GEXSCOPE, BD Rhapsody™, and fluidigm). Spatial transcriptomics was performed using 10× Genomics Visium or Stereo-seq (STOmics Gene Expression). A large variability exists regarding the number of recovered cells, genes identified per cell, and sequencing depth (Table 1 and Supplementary Table S2). A minimum of 43 and maximum around 10 000 cells/placenta have been captured, with expressed genes/cell ranging from 340 to 4848 across all studies. For placental 10× analyses, a positive linear regression applies to the relation between the number of reads (i.e. depth of sequencing) and the number of genes identified per cell (*R*^2^ = 0.52, *P* < 0.01, Fig. 1a), showing that deeper sequencing yields a greater number of genes/cell. An exponential one-phase decay regression can also be applied between the number of recovered cells and the depth of sequencing (Fig. 1b). This relation, however, is weaker (*R*^2^ = 0.30), suggesting that increasing the sequencing depth does not guarantee more cells for downstream analyses, as determined by initial sample quality (Maitra *et al.*, 2021). These results suggest that future scRNAseq studies need to optimize placental sample collection, as already recommended (Burton *et al.*, 2014), to maximize the number of recovered cells. The recommended depth of sequencing between 30 000 and 70 000 for 10× Genomics provides sufficient information for the downstream analyses but increased sequencing depth to 250 000 reads/cell is still below the saturation.

### Single-nucleus RNA sequencing

snRNAseq is a method recently derived from the scRNAseq protocol. The main difference from scRNAseq, which requires fresh tissue with good quality and ability to dissociate cells, is that snRNAseq allows analysis of frozen or difficult-to-dissociate tissues. However, although scRNAseq measures both cytoplasmic and nuclear transcripts, snRNAseq only measures nuclear transcripts. In contrast to cytosolic RNAs, nuclear RNAs represent only 10–15% of the total RNAs (Barthelson *et al.*, 2007), and most are nascent RNAs before splicing (Zaghlool *et al.*, 2013). The distribution of protein coding genes is comparable, but the abundance of certain genes differs between two cellular compartments. Mitochondrial genes are also more abundant in cytosol (Zaghlool *et al.*, 2021) and long non-coding RNAs, and small nucleolar RNAs are enriched in the nucleus (Mas-Ponte *et al.*, 2017). Considering these differences, care should be taken when analyzing data and interpreting the results. Moreover, after mapping, more than 80% of reads from cytosolic RNAs are in exonic regions compared to only 20% in nuclear RNAs. Therefore, in snRNAseq, the inclusion of introns is an important mapping criterion, while in scRNAseq, it is less of a determinant. Furthermore, it has been advised to use a 10–15% threshold for mitochondrial content to determine good quality cells in scRNAseq, but this limit must be lowered for snRNAseq as mitochondrial material primarily come from cytosol and is part of the ambient RNA in this technique.

So far, three studies have reported on the use of snRNAseq (Cao *et al.*, 2020; Pique-Regi *et al.*, 2020; Arutyunyan *et al.*, 2023). The first study achieved the collection of around 2700 nuclei/sample with around 340 genes/nucleus using sci-RNA-seq3 and shallow sequencing depth (Cao *et al.*, 2020). The second study used 10× on pooled placenta and decidua from 32 individuals, resulting in only 415 nuclei/sample with a mean of 510 genes/nucleus (Pique-Regi *et al.*, 2020). More recently, Arutyunyan *et al.*, published snRNAseq data from both placenta and decidua from first trimester samples and showed a higher nuclei recovery and quality (180 721 nuclei from five donors with a mean of 1791 genes/nucleus, similar to 1871 genes/cell obtained using scRNAseq), although it should be noted that this result includes peripheral blood and myometrium. In other tissues, a similar sensitivity of gene detection was observed in snRNAseq compared to scRNAseq, although slightly different cell compositions were observed (Wu *et al.*, 2019; Slyper *et al.*, 2020). This demonstrates that snRNAseq could be an appealing method for future human placenta studies, considering the logistical and planning advantages of using frozen tissue.

Besides snRNAseq, Arutyunyan *et al.*, (2023) also performed chromatin accessibility analysis through single-nucleus ATAC-sequencing to compare the cell types in trophoblast invasion. This is the first single-nucleus multi-omic analysis performed on human placenta, and only one other study has performed single-cell ATAC-sequencing on the human placenta, where samples collected at 12–18 weeks of pregnancy were analyzed (Domcke *et al.*, 2020), although this study lacks further details about placenta analysis.

Considering the increasing number of studies using snRNAseq and/or scRNAseq on human placentas, a consensus regarding sample collection and data quality is necessary to better understand this organ and its role in pregnancy pathologies. Data sharing is also essential to improve the quality of the research on human placenta. For instance, in the human cell atlas (HCA), at the time of this review, only three studies with a total of 27 donors are included for placenta, while other organs, such as the lung and kidney, have been analyzed using single-cell technologies in more than 400 donors (Regev *et al.*, 2017). Furthermore, these studies refer to first trimester placentas only and there are no data for later stages of human placenta development.

### Bioinformatic analysis

#### Literature-mining databases

Apart from individual studies, literature-mining efforts have aggregated information into databases regarding placental cell diversity and signature expression. In CellMarker, marker genes are reported for 17 cell types from human placentas (Hu *et al.*, 2022). The human protein atlas reanalyzed the first trimester data from Vento-Tormo *et al.* for inclusion in their new version (Karlsson *et al.*, 2021). Cao *et al.* deposited their data and DEG list in Descartes cell atlas (https://descartes.brotmanbaty.org/bbi/human-gene-expression-during-development/). The Human BioMolecular Atlas Program (HuBMAP) ASTBplusB tables include information about full-term human placenta based on curated protein and lipid biomarkers for 24 cell types from published placental histopathologic nomenclature, and literature on cell-type-specific biomarkers (Laurent *et al.*, 2022). These databases can assist in annotation of clusters in human placenta but not all placental cell types from different GAs have been included in these databases. Therefore, the curation of the appropriate markers is mandatory for current and future studies.

#### Quality control for the single-cell datasets

In sc- and sn-RNAseq, cell quality must be confirmed before the downstream analyses, especially in droplet-based methods where empty droplets containing ambient RNAs or droplets containing two or more cells/nuclei exist. Only 21 studies reported filtering criteria for poor-quality cells (Supplementary Table S3). Most have used a threshold for the quantity of genes and/or unique molecular identifiers (UMIs) recovered and the mitochondrial content per cell. However, these thresholds varied from 100 to 3000 genes-UMI/cell and 10–25% of mitochondrial content/cell. Furthermore, several studies defined gene expression by at least three cells, but some did not use the same threshold and others did not report any threshold. Only two studies tried to remove the ambient RNAs using a list of genes often found in ambient RNA (Vento-Tormo *et al.*, 2018) or filtering out genes with a low fraction of reads in cells (Arutyunyan *et al.*, 2023).

To remove doublets, some studies filtered out cells with more than 6000 genes (Supplementary Table S3). Manual identification has been performed based on the expression of multiple lineage markers. However, in the more recent studies, packages such as DoubletFinder (McGinnis *et al.*, 2019), Scrublet (Wolock *et al.*, 2019), or scDblFinder (Germain *et al.*, 2022) were often used to identify and remove doublets.

#### Definition of cell-type markers

To identify new cell-type markers, most studies used the FindMarkers function of the Seurat R package with different statistical methods. Only 21 studies reported the method used to define new signature genes (Supplementary Table S3). The most common method was the Wilcoxon rank-sum test. The likelihood ratio test, area under receiver operating characteristic curve analysis, MAST GLM (Model-based Analysis of Single Cell Transcriptomics Generalized Linear Model)-framework using cellular detection rate as a covariate, statistical methods based on the negative binomial distributions, z-score-based methods, and threshold of fold-change of mean expression values were also used. The rationale behind the use of different methods is likely adaptation to the data (i.e. the number of cells of each type, the number of overlapping genes between the output DEG list, and the established cell type markers).

As shown, sc- and snRNAseq studies have used different pipelines to assess good-quality cells and define marker genes. However, several reviews provided detailed information to advise the handling of scRNAseq data adapted to each different dataset (Balzer *et al.*, 2021; Zhang *et al.*, 2021b; Ke *et al.*, 2022). These differences have also highlighted the complexity involved in data integration from sc- and snRNAseq.

### Systematic review: placental cell type annotation

**Figure 2.**
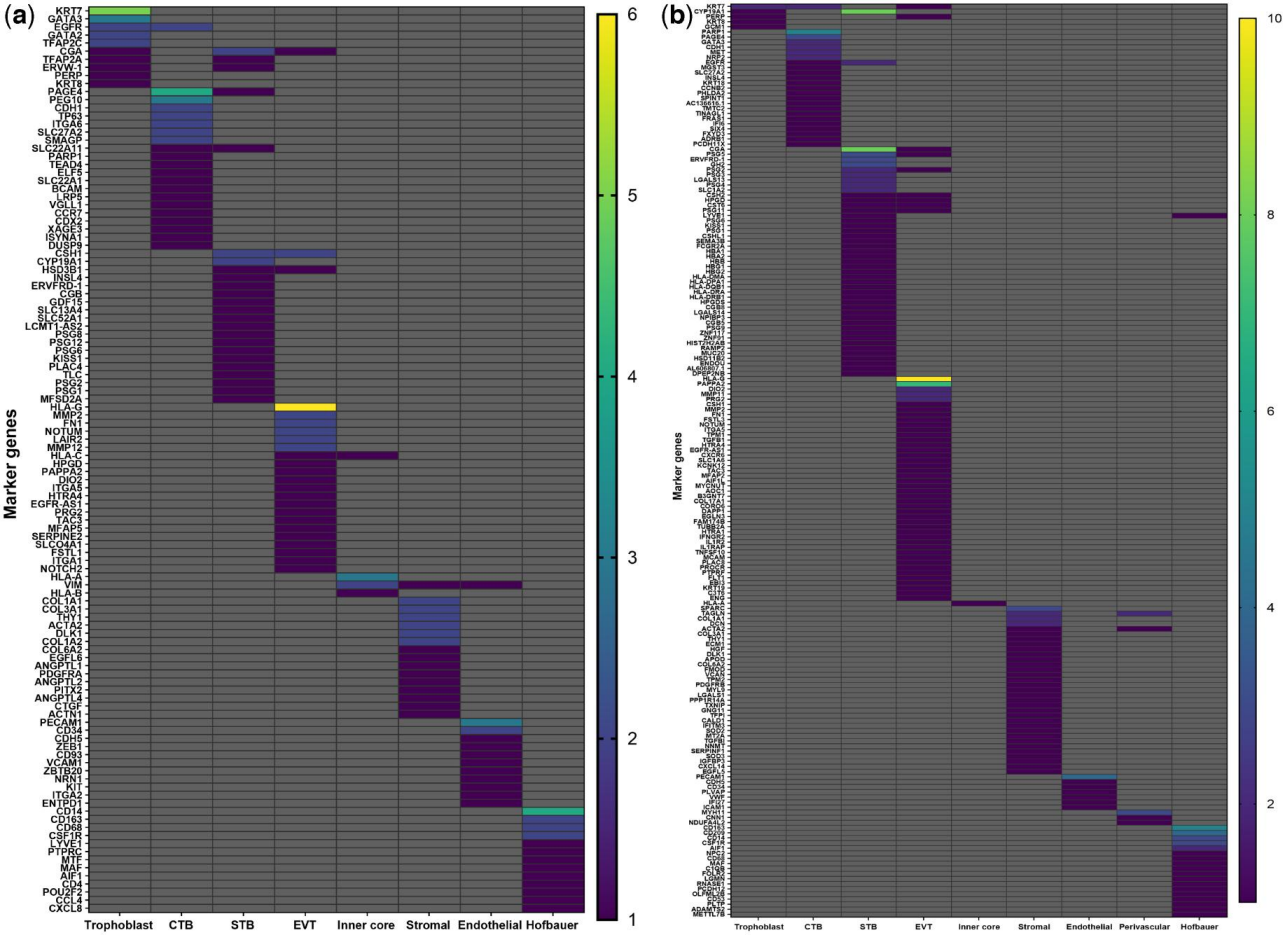
**Heatmap of commonly used genes to define the different human placental cell types from first trimester and term.** Analysis of first trimester (**a**) and term (**b**) literature on single-cell RNA sequencing of human placenta. The scale bar represents the occurrence of the gene as a marker to define placental cell types in the different single-cell RNA sequencing studies on human placenta. Only genes that have been used more than once are represented. The complete list is in Supplementary Table S2. CTB: cytotrophoblast; EVT: extravillous trophoblast; STB: syncytiotrophoblast.

For each study, the defined cell types and subtypes vary, as summarized in Table 2. The markers used to annotate each trophoblast type also vary between the studies (Fig. 2; Supplementary Table S4). One striking observation is that no specific marker is shared by all studies for any cell type, regardless of the GA and cell type. Furthermore, several genes that are defined as markers in several studies are sometimes used to define another cell type in a different study (Supplementary Table S4). Moreover, most of the newly defined markers in one study are not applicable to other studies. However, by comparing the markers used, we were able to identify genes commonly used in several studies to define the same cell type. Most of these were used regardless of the GA, but some were used to define only one cell type at a specific GA (Supplementary Table S4).

**Table 2.** Fetal cell types defined in the different studies using single-cell RNA sequencing of the human maternal–fetal interface at different stages of pregnancy.

		Total number of cells	Method of analysis	Number of clusters	Trophoblasts			Inner core cells		Fetal vessels	
					CTB	STB	EVT	Mesenchymal stromal cells/fibroblasts	Hofbauer cells	Perivascular cells	Endothelial cells
First trimester (<13 weeks)	(Suryawanshi *et al.*, 2018)	14 341	Single cell, 10× Genomics and Drop-seq	9	Yes	Yes	Yes	3	Yes		Yes
	(Liu *et al.*, 2018b)	1471 (first and second trimester placentas)	Single cell, FACS and SMART-seq2	4	3	Yes	3	2	2	NA	NA
	(Vento-Tormo *et al.*, 2018)	18 547	Single cell, SMART-seq2 and 10× Genomics	29 in total (9 of fetal cells)	2	Yes	2	2	Yes		Yes
	(Sun *et al.*, 2020)	7245	Single cell, 10× Genomics	5	Trophoblasts in general			Yes	Yes		Yes
	(Han *et al.*, 2020)	19 493	Single-cell, Microcell-seq	19	2	yes	3	3	Yes	Yes	
	(Saha *et al.*, 2020)	–	Single cell, 10× Genomics	22	Yes	Yes	Yes	Defined as non-trophoblast cells			
	(Du *et al.*, 2021)	66 078	Single cell, 10× Genomics	26 (1 of fetal cells)	NA	NA	yes	NA	NA	NA	NA
	(Shannon *et al.*, 2022)	50 790 (including data from Vento-Tormo *et al.*, 2018 and from organoids)	Single cell, 10× Genomics	31	6	Yes	Yes	6	Yes	Yes	Yes
	(Zheng *et al.*, 2022)	218 899	Single cell, 10× Genomics	18 maximum	1 or 2	1 or 2	1 or 2	Yes	1–3	Yes	0–2
	(Liu *et al.*, 2022)	–	Stereo-seq, STOmics Gene Expression	7	Yes	Yes	Yes	2	Yes		Yes
	(Pan *et al.*, 2022)	63 249	Single cell, BD Rhapsody™	20	Yes	No	Yes	Mixed with maternal cells			
	(Arutyunyan *et al.*, 2023)	319 740 cells and 180 721 (+54 513 in multiome) nuclei (including peripheral blood and myometrium)	Single cell and single nuclei, 10× Genomics RNA and multiome + spatial transcriptomics, 10× Genomics Visium	19 (13 of fetal cells)	4	Yes	5	Yes	Named fetal myeloid		Yes
Second trimester (13–26 weeks)	(Liu *et al.*, 2018b)	1471 (first and secnd trimester placentas)	Single cell, FACS and SMART-seq2	1	NA	NA	2	NA	NA	NA	NA
	(Pique-Regi *et al.*, 2020)	13 190	Single cell, 10× Genomics	19	2	Yes	Yes	3 fibroblast and stromal cells	Yes	3 fibroblast and stromal cell subtypes	
	(Cao *et al.*, 2020)	29 876	Single nuclei, Sci-RNA-seq3	12	Yes		Yes	Yes	In myeloid cells?	In smooth muscle cells?	Yes
	(Marsh *et al.*, 2022)	50 496	Single cell, 10× Genomics	13	6	2	4	In stromal cells	In immune cells?	In stromal cells	
Term (>26 weeks)	(Pavličev *et al.*, 2017)	87	Single cell, Fluidigm C1 and laser	5	3	Yes	Yes	NA	NA	NA	NA
	(Tsang *et al.*, 2017)	20 518	Single cell, 10× Genomics	12	Yes	Yes	Yes	Yes	Yes	Yes	Yes
	(Pique-Regi *et al.*, 2019)	77 906	Single cell, 10× Genomics	19	2	Yes	Yes	3 fibroblast and stromal cells	Yes	3 fibroblast and stromal cell subtypes	
	(Pique-Regi *et al.*, 2020)	13 305 cells and nuclei	Single cell, 10× Genomics	19	2	yes	yes	3 fibroblast and stromal cells	Yes	3 fibroblast and stromal cell subtypes	
			Single nuclei, 10× Genomics		1	2	yes	2 fibroblast and stromal cells	Yes	2 fibroblast and stromal cell subtypes	
	(Huang *et al.*, 2020)	29 231	Single cell, 10× Genomics	16 (5 of fetal cells)	NA	NA	5	NA	NA	NA	NA
	(Lu-Culligan *et al.*, 2021)	44 140	Single cell, 10× Genomics	21 (including decidual origin)	Yes	Yes	Yes	Yes	Yes		
	(Zhang *et al.*, 2021a)	11 518	Single cell, GEXSCOPE	13	Yes	Yes	Yes	Yes	Named macrophages	Yes	Yes
	(Yang *et al.*, 2021)	27 220	Single cell, 10× Genomics	15	9	3	5		Named macrophages		
	(Zhou *et al.*, 2022)	29 008 (including controls from Yang *et al.*, 2021)	Single cell, 10× Genomics	18	3	Yes	4	Yes	Named macrophages		
	(Zhang *et al.*, 2022)	27 607 (including controls from Yang *et al.*, 2021)	Single cell, 10× Genomics	18	4	Yes	2		Named macrophages		
	(Chen *et al.*, 2022)	17 481	Single cell, 10× Genomics	20	3	Yes	Yes	2	Yes	Yes	Yes
	(Garcia-Flores *et al.*, 2022)	–	Single cell, 10× Genomics	18	2	Yes	2	4 fibroblast and stromal cells	2	4 fibroblast and stromal cells	
	(Wang *et al.*, 2022)	11 438	Single cell, 10× Genomics	27	6	Yes	2	7	In immune cells?	4 subtypes	2 subtypes
	(Campbell *et al.*, 2023)	9244	Single cell, 10× Genomics	19 fetal clusters	2	Yes	Yes	Yes	Yes		Yes

In trophoblasts, inner core cells and fetal vessels, a number represents the number of cell subtypes or states defined as a cluster of cells on UMAP (Uniform Manifold Approximation) or t-SNE (t-distributed stochastic neighbor embedding) analysis according to the study. Yes, means that this cell type had been identified in the study without defining subtype. NA means that this cell type has been removed during the sampling or the analysis.

CTB: cytotrophoblast; EVT: extravillous trophoblast; STB: syncytiotrophoblast.

#### Trophoblasts

**Figure 3.**
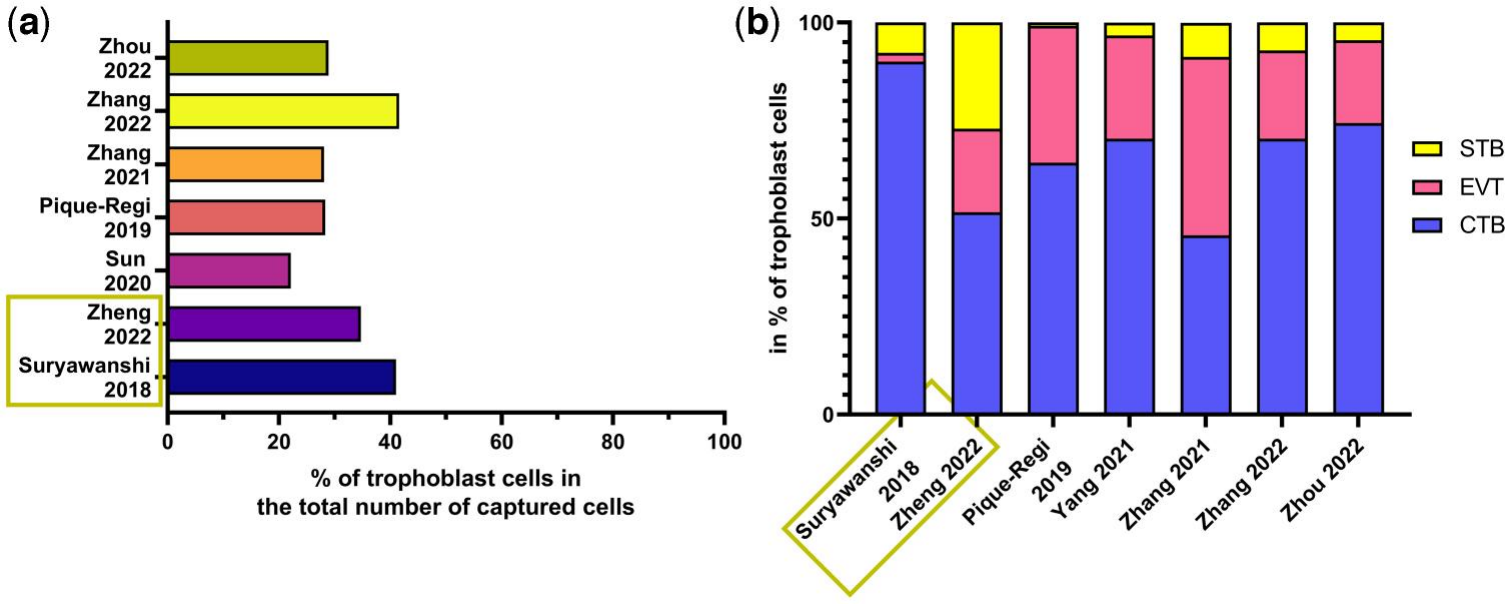
**Proportion of cells reported in studies of single-cell RNA sequencing in human placenta.** (**a**) Proportion of trophoblasts out of all captured cells and (**b**) proportion of each of the three kinds of trophoblast cells on the total of trophoblast. CTB: cytotrophoblast; EVT: extravillous trophoblast; STB: syncytiotrophoblast. Only Suryawanshi *et al.* (2018) and Zheng *et al.* (2022) reported the proportion of trophoblast cells of placentas from the first trimester and are highlighted in the green boxes; the remaining studies were performed on term placentas.

Several studies reported a proportion of 20–42% of cells identified as trophoblasts, regardless the GA (Fig. 3a; Suryawanshi *et al.*, 2018; Pique-Regi *et al.*, 2019; Sun *et al.*, 2020; Zhang *et al.*, 2021a, 2022; Zheng *et al.*, 2022; Zhou *et al.*, 2022). Except for one study (Pan *et al.*, 2022), the three trophoblast types are always identified but, when reported, the proportion of each type ranges widely between studies (Fig. 3b). The sampling and dissociation methods could contribute to these differences as three studies obtained comparable proportions of each trophoblast type when using the same methods (Yang *et al.*, 2021; Zhang *et al.*, 2022; Zhou *et al.*, 2022). However, owing to insufficient information, especially on the sampling site in most of the studies, it is difficult to conclude which factor affects this composition the most.

To define trophoblast cells, *KRT8* and *PERP* were commonly used between studies, regardless of GA, while *GATA2* and *TFAP2C* were also used, but only for the first trimester placenta analysis (Supplementary Table S4). In addition, *KRT7* and *GATA3* were the frequently used markers to define trophoblast cells in the first trimester, whereas in term placentas, only *KRT7* was used in more than one study (Fig. 2).

Many genes used to define CTBs were also often used for STBs (Supplementary Table S4), showing that characterization of CTBs from STBs is suboptimal in current scRNAseq studies. To define CTBs, *PAGE4* and *PEG10* for the first trimester and *PAPR1* were the most used marker genes (Fig. 2). Considering genes used only to define CTBs, *SLC27A2* and *CDH1* were used in at least two studies regardless of GA (Supplementary Table S4). For the first and second trimesters, *SMAGP*, *TP63*, *PEG10*, and *ITGA6* were commonly used for only CTB, and *NRP2* was the only gene used for term placentas (Supplementary Table S4).

For STB, *CGA* and *CYP19A1* are the most used genes for both first trimester and term analyses (Fig. 2). *CSH1* was also the most used marker gene to define STB in first trimester analysis, but it was used by several studies to define EVT as well (Fig. 2). *ERVFRD1*, *PSG1*, *PSG6*, and *KISS1* were the common genes used to define only STBs, regardless of GA (Supplementary Table S4). In the term placenta, *PSG3*, *PSG4*, *GH2*, *LGALS13*, and *SLC1A2* were also commonly used to only define STB (Supplementary Table S4).

*HLA-G* is the most often used EVT marker regardless of the GA but was not used in all studies (Fig. 2). In term placentas, *PAPPA2* was used by seven studies to define EVT. Altogether, 14 genes were commonly used to define only EVTs. Most of them were used regardless of GA (*DIO2*, *PAPPA2*, *HTRA4*, *PRG2*, *EGFR-AS1*, *ITGA5*, *TAC3*, *NOTUM*, *MMP2*, and *FN1*) but interestingly *LAIR2*, *MFAP5*, and *HPGD* were only used in the first and second trimester placenta analyses and *MMP11* was only used to define EVTs in the term placenta.

#### Cytotrophoblasts

CTBs are always the most represented trophoblast type in scRNAseq analyses. Their proportion varies from 45% to 90% of total trophoblast cells across studies, regardless of the GA (Fig. 3b). Multiple subtypes of CTBs have been identified in most published studies (Pavličev *et al.*, 2017; Liu *et al.*, 2018b; Vento-Tormo *et al.*, 2018; Pique-Regi *et al.*, 2019; Han *et al.*, 2020; Pique-Regi *et al.*, 2020; Yang *et al.*, 2021; Marsh *et al.*, 2022; Shannon *et al.*, 2022; Wang *et al.*, 2022; Zhang *et al.*, 2022; Zhou *et al.*, 2022; Arutyunyan *et al.*, 2023).

The proliferative CTB subtype is commonly identified using markers such as *MKI67* (Vento-Tormo *et al.*, 2018; Marsh *et al.*, 2022; Wang *et al.*, 2022; Zhang *et al.*, 2022; Arutyunyan *et al.*, 2023), *YAP1*, *SPINT2*, *MSI*, *BCAM* (Shannon *et al.*, 2022), *PEG10* (Han *et al.*, 2020), *RRM2*, *CCNB1*, *CDNK1* (Liu *et al.*, 2018b), *PNCA*, and *CDK1* (Chen *et al.*, 2022) or *STMN1* (Campbell *et al.*, 2023). One study further characterized proliferative CTB subtypes in S-phase, expressing *PCNA*, and in G2/M phase with the expression of *TOP2A* (Marsh *et al.*, 2022). The proliferative subtype has been identified at different GAs, confirming CTB proliferation throughout the pregnancy in humans. In term placentas, however, they were more abundant in the middle section of placenta compared to the maternal and fetal sides (Wang *et al.*, 2022).

Most studies identified at least one non-proliferative class of CTB, often characterized by a high expression of *PAGE4* (Pique-Regi *et al.*, 2019; Han *et al.*, 2020; Marsh *et al.*, 2022; Zhang *et al.*, 2022) but few studies defined further CTB subtypes. Liu *et al.* (2018b) identified a CTB subtype in the first trimester placenta that seems to be ready to fuse with the STB as it expresses high levels of Syncytin-2, and another non-proliferative CTB subtype but which does not express Syncytin-2. Also using the first trimester placentas, Shannon *et al.* defined a total of five subtypes in addition to the proliferative CTBs, allowing the distinction of vCTBs from CCCs. Moreover, they identified three vCTB subtypes (respectively, *TEAD4*+, *ELF5*+, and *EGFR*+) and, using trophoblast organoids, they postulated that these correspond to possible different physiological states rather than different subtypes. They also observed two subclasses of CCCs: one that seemed to be the progenitor cells for EVT expressing high levels of *ITGA2*, *SOX9*, and *NOTCH1*, migrating from the proximal zone of the anchoring column; the other with high expression of *ITGA5*, *NOTCH2*, and *HLA-G* that seems to be an intermediate state between the first CCC subtype and EVTs (Shannon *et al.*, 2022). Arutyunyan *et al.*, (2023) defined three CTB subtypes in the first trimester placentas in addition to the proliferative subtype and were able to precisely localize them in the placental villi and the trophoblast shell, using spatial transcriptomics combined with scRNAseq and snRNAseq. One cluster was localized in the placental villi area, beneath the STB layer corresponding to vCTBs. Both vCTBs and proliferative CTBs highly expressed *TP63*, *CDH1*, and *BCAM* as well as additional stem and progenitor cell markers such as *LGR5* and *L1TD1*, Wnt signaling molecules (*WLS* and *TNIK*), and SEM3F-NRP2 signaling complex. In the same area, they also identified fusing-CTBs, an intermediate state between CTB and STB, which showed down-regulated expression of WNT and BMP signaling (*BMP7*) genes and up-regulated expression of endogenous retroviral genes (*ERVW-1*, *ERVFRD-1*, and *ERVV-1*). The last subtype was observed in the trophoblast shell at the junction of the placenta and the decidua, and corresponded to an intermediate state between CTB and EVT. Similar to the fusing-CTBs, Wnt and bone morphogenetic protein (BMP) signaling were again down-regulated; however, this pre-EVT CTB subtype also up-regulated *NOTCH1*, *ITGB6*, *ITGA2*, and *LPCAT1* (Arutyunyan *et al.*, 2023).

In term placentas, CTBs from the fetal side were involved in regulation of cell activity, CTBs from the middle section in nutrient and gas exchange, and those from the maternal side were participating in regulation of the inflammatory response and vascular development (Wang *et al.*, 2022). In a similar manner, using the chorionic plate from the second trimester placentas, Marsh *et al.* defined one CTB cluster highly expressing *PAGE4*, *PEG10*, and *CDH1*, as well as three other clusters that expressed intermediate levels of these genes, suggesting various states of a common differentiation pathway. They also demonstrated that a CTB subtype highly expressing cytokeratin was only present on the side of the villous tree and not in the chorion under the villous tree (Marsh *et al.*, 2022). Using the early third trimester placentas (28–30 weeks), Chen *et al.* (2022) characterized one cluster that highly expressed *SERPINE1* and *NEAT1*, *GCM1*, *SLC1A5*, and *FZD5* engaged in the syncytial pathway and another cluster that highly expressed *HSD17B1* and *PHLDA2* involved in hormone biosynthesis.

Using snRNAseq, Pique-Regi *et al.*, recovered a small amount of CTBs but increased the number of STBs captured compared to the scRNAseq studies. Furthermore, they did not identify non-proliferative CTBs while using the same cell type annotation as their previous scRNAseq study (Pique-Regi *et al.*, 2019, 2020). Arutyunyan *et al.* (2023) also performed both scRNAseq and snRNAseq and did not observe any reduction in CTBs captured in snRNAseq. However, Pique-Regi *et al.* (2020) demonstrated the possibility of identifying CTBs at different GAs using the same markers.

Using the trajectory analysis, CTBs in the first trimester exhibited two possible ways of differentiation that lead to the same EVT endpoint (Shannon *et al.*, 2022). More recently, Arutyunyan *et al.* focused on the trajectory of EVTs in the first trimester placenta, considering not only iEVTs and eEVTs but also GCs. All EVT subtypes were shown to originate from CCCs that were derived from CTBs (Arutyunyan *et al.*, 2023). In contrast, in term placenta cells expressing high levels of cell-cycle-related genes formed minor branches along the EVT path, suggesting that some CTBs in the process of differentiation into EVTs re-enter the cell cycle. The STB branch, on the other hand, bifurcated from CTBs into one sub-branch with cells expressing gestational hormone genes and genes involved in cell fusion (Tsang *et al.*, 2017).

#### Syncytiotrophoblast

Even though the STB layer covers placental villi, the number of recovered STBs is relatively small in all published scRNAseq studies (Fig. 3b). The large size of this multinucleated cell is suboptimal for scRNAseq; hence it is logical that this cell type is not well captured in single-cell dissociations of placenta. Cells identified as STB in scRNAseq data are likely precursors of STB and look like CTB at the initial stages of cell fusion. Indeed, *ERVFRD-1* was shown using spatial transcriptomics and snRNAseq to be a marker for fusing-CTBs and not of established STB (Arutyunyan *et al.*, 2023). This gene is commonly used in studies to define STB, suggesting the possible incorrect annotation of this cell type in the previous scRNAseq studies. The use of snRNAseq has further demonstrated that STB is not properly captured in scRNAseq (Pique-Regi *et al.*, 2020; Arutyunyan *et al.*, 2023).

Taken together, there are discrepancies in the classification and capture of STB in previously published studies. One study was able to identify three STB clusters that were separated by their expression of *ENDOU*, *DHRS9*, *POU2F2*, *SPIDR*, *S1PR2*, and *ECI2* but did not further characterize them (Yang *et al.*, 2021). Using snRNAseq, two clusters of STB were identified, but again without further investigation (Pique-Regi *et al.*, 2019). The most recent snRNAseq study only identified one big cluster of STB nuclei (Arutyunyan *et al.*, 2023). Therefore, the identity and possible subtypes of STB is not entirely clear and needs to be further elucidated.

#### Extravillous trophoblasts

In scRNAseq studies on term placentas, EVTs are the second most recovered trophoblast type, ranging from around 22–45%, while in the first trimester, EVTs accounted only for 2–21% of the trophoblast cells (Fig. 3b). As EVTs differentiate later in the gestation compared to CTBs and STBs, it seems logical to observe less EVTs in the first trimester placenta. Studies have shown that EVTs can be found in all parts of the placenta, from the chorionic plate to the decidua, regardless of GA. They were, however, found in higher numbers in the middle/placental villi region of the placenta (Pique-Regi *et al.*, 2019; Marsh *et al.*, 2022; Wang *et al.*, 2022).

EVT subtypes have been identified, but several studies defined them only with a list of genes (Han *et al.*, 2020; Yang *et al.*, 2021; Wang *et al.*, 2022; Zhang *et al.*, 2022). In the first trimester placenta, a proliferative EVT subtype was observed in both villi and decidua sections (Liu *et al.*, 2018b; Vento-Tormo *et al.*, 2018; Arutyunyan *et al.*, 2023). Using spatial transcriptomics, these proliferative EVTs were shown to co-localize with CCCs in the trophoblast shell of anchoring villi (Arutyunyan *et al.*, 2023). Liu *et al.* (2018b) observed that they highly express *RRM2*, important for DNA replication (). In the second trimester, however, the proliferative type was not identified in the chorionic plate (Marsh *et al.*, 2022) and was not clearly identified either in the term villous tree (Zhou *et al.*, 2022). However, in term placentas, proliferative EVTs were present in decidua and showed upregulated *INSL4* expression (Huang *et al.*, 2020).

Liu *et al.* also identified two other EVT subtypes localized at the proximal end of the CCCs in the first trimester placenta. One of the subtypes expressed genes involved in receptor activation regulation and immune response (i.e. *TAC3*, *SERPINE*, *PRG2*, and *JAM2*), whilst the other subtype seems to be an intermediate of the proliferative state and the non-proliferative state (Liu *et al.*, 2018b). The first subtype was demonstrated to be a precursor of EVTs and presented strong similarities with EVTs identified in the placentas of the second trimester. At this later gestational stage, the authors also defined two EVT subtypes, one once again related to immune response and the other related to growth regulation and gonadotrophin secretion (Liu *et al.*, 2018b).

Also using the first trimester placentas, Arutyunyan *et al.* (2023) defined four more EVT subtypes using both snRNAseq and spatial transcriptomics. The first subtype was the bifurcation point between iEVTs and eEVTs and was localized at the distal end of anchoring villi in the trophoblast shell area. They also identified eEVTs with up-regulation of *NCAM1*, *GGT1*, *PPFIA4*, and *MMP12* in the spiral arteries and iEVTs highly expressing *PLAC8*, *SERPINE1*, and *SERPINE2* and down-regulating *PLAU*. Interestingly, they are the only group who identified GC, with up-regulation of *RAC1*, *CD81*, and the PRG2–PAPPA complex (Arutyunyan *et al.*, 2023).

In the second trimester, EVTs were identified in the chorionic plate area, under and around the villous tree, but while EVTs under the villous tree were observed deep in the decidua, the ones from the side remained in the epithelial layer (Marsh *et al.*, 2022). Four clusters were identified in both areas; however, one subtype expressing *CGM1*, *PPARG*, and *CEBPB*, showing enrichment for placental development and antigen presentation, was twice as abundant under the villous tree compared to the surrounding zone, likely corresponding to newly differentiated EVTs. In contrast, all other subtypes were half as abundant under the villous tree and were enriched in genes related to extracellular structure, matrix organization, glycosylation, and peptidase activity (Marsh *et al.*, 2022).

In term placentas, Zhou *et al.* defined a total of four subtypes of EVTs classified as important for: cell invasion and immunity; or protein processing and synthesis; or cell migration, chromatin modification, and morphogenesis; or the mRNA metabolic process, intrinsic apoptotic signaling pathways, and oxidative phosphorylation (Zhou *et al.*, 2022). In the decidual compartment, Huang *et al.* (2020) identified four clusters in addition to a proliferative one characterized by up-regulation of *FABP7*, *ALPP*, *DSG1*, or *PSG2* and varying expression of *HLA-G*.

#### Meta-analysis of already annotated datasets

##### Method

Given the discrepancy of marker genes identified across studies, we reanalyzed published expression matrices with already annotated cell types, considering the GA. For first trimester, the available data with annotated cell types were downloaded and included (from four publications: Liu *et al.*, 2018b; Suryawanshi *et al.*, 2018; Vento-Tormo *et al.*, 2018; Shannon *et al.*, 2022). Only two studies provide annotated gene count matrices of placental single-cell/nuclei transcriptomics in term placentas (Pique-Regi *et al.*, 2019; Yang *et al.*, 2021). Owing to discrepancies in the definition of the non-trophoblast cell types, we focused on trophoblast types (CTB, STB, and EVT). After log normalization, Wilcoxon rank-sum tests on all cells (adjusted *P*-value <0.05 and fold-change threshold >2 or 4, according to the data) were performed to gain hundreds of DEGs from each study. We then used UpSet diagrams to represent the results from this analysis.

##### Results

**Figure 4.**
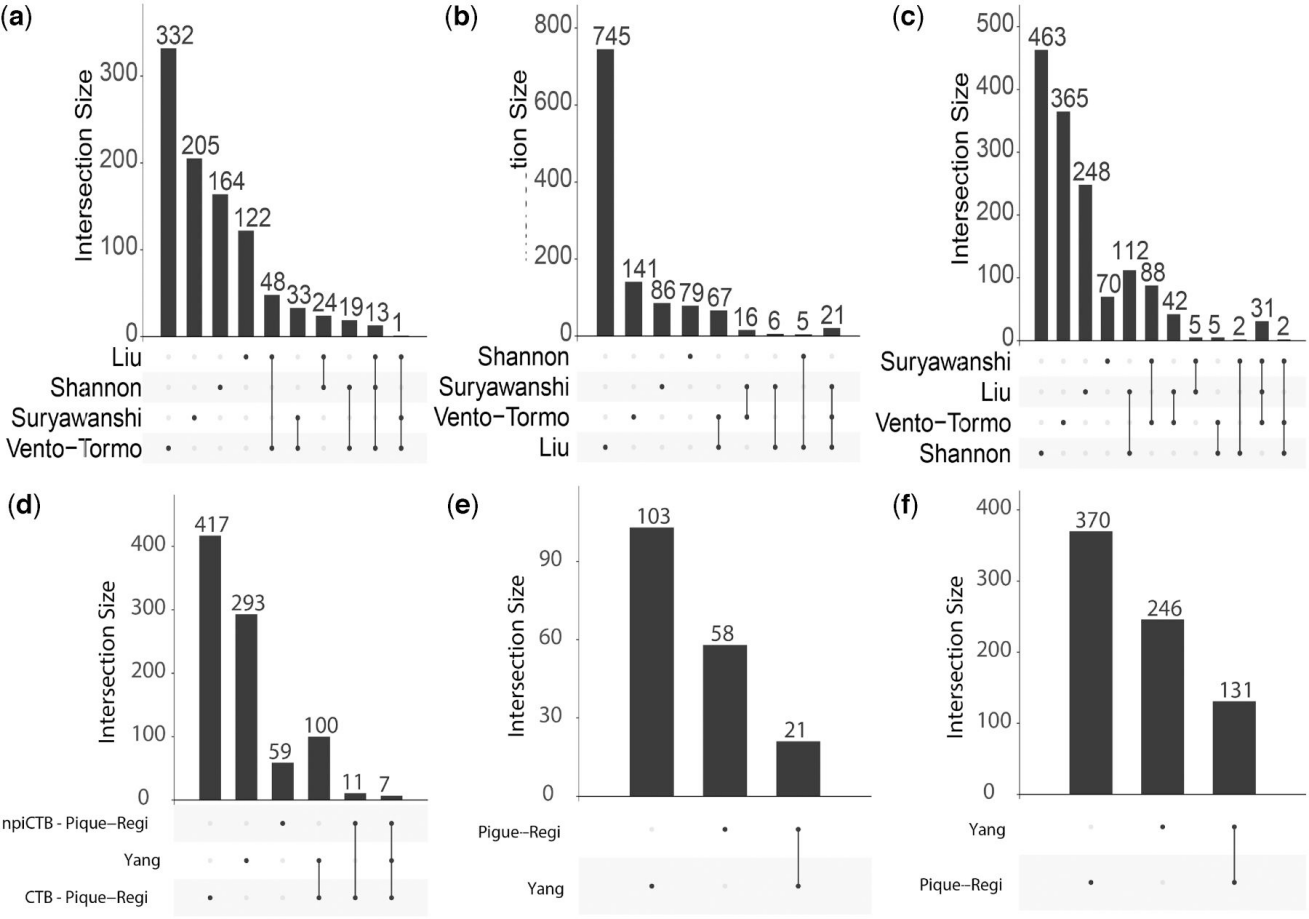
**Upset plots of differentially expressed genes in the different trophoblast types by comparing original count matrices of studies on first trimester and term human placenta**. (**a**) and (**d**) CTB, (**b**) and (**e**) STB, (**c**) and (**f**) EVT differentially expressed genes. (a–c) correspond to analysis of literature on first trimester analyses and (d–f) on term analyses. DEGs have been obtained using Wilcoxon rank-sum tests on all cells in each study and using thresholds on the *P*-adjusted values (*P*-adj < 0.05) and the fold-change >2 or 4 according to the study. UpSet plots show intersections between gene sets from each study in a matrix, with the rows of the matrix corresponding to the gene sets, and the columns to the intersections between genes sets from these studies. The size of the gene sets and of the intersections are shown as bar charts. First author’s names are displayed as set names for first trimester studies (Liu *et al.*, 2018b; Suryawanshi *et al.*, 2018; Vento-Tormo *et al.*, 2018; Shannon *et al.*, 2022) and term (Pique-Regi *et al.*, 2019; Yang *et al.*, 2021). CTB: cytotrophoblast; DEGs: differentially expressed genes; EVT: extravillous trophoblast; STB: syncytiotrophoblast.

In first trimester placentas, most of the DEGs for each trophoblast type were not shared among studies (Fig. 4a). Of greater concern, no gene, irrespective of the subtype of trophoblast, was identified by all studies as a DEG among trophoblast cell types. Nevertheless, 14, 21, and 33 genes were common in at least three studies for CTB, STB, and EVT, respectively.

Although less studies were available in term placentas for this analysis, a similar observation as for the first trimester was made: most DEGs are not shared between studies, regardless of the trophoblast type (Fig. 4d–f). Furthermore, the definition of CTB type differed between the two studies: Pique-Regi *et al.* (2019) identified CTB and less abundant non-proliferative interstitial CTB (npiCTB), while Yang *et al.* (2021) identified only one vCTB type. Of particular interest is that 18 genes are shared between both CTB subtypes defined by Pique-Regi *et al.* (Fig. 4), demonstrating that their method failed to properly distinguish these transcriptionally similar cell types, compared to other placental cell types. The vCTB from Yang *et al.*, however, showed 100 highly expressed genes shared with CTB, and only seven with the npiCTB of Pique-Regi *et.al.* (Supplementary Table S5). The STB and EVT in the two studies shared 21 and 131 genes, respectively (Supplementary Table S5).

### Fetal non-trophoblast cells

The fetal non-trophoblast cells are less frequently described in scRNAseq studies. In most publications, the cells are often grouped in generic types, such as fibroblast and stromal cells, the latter encompassing endothelial cells, perivascular cells, and macrophages (Table 2).

Most studies using placentas of different trimesters have used *HLA-A* as a marker of non-trophoblast cells (Fig. 2) but the definition of these cells is variable between studies. In term placentas, *SPARC* was the most used marker to define stromal cells (Fig. 2b). Common genes to define stromal cells, regardless of the GA, are *COL1A1*, *COL3A1*, *COL6A2*, *DCN*, and *THY1*, while *COL1A2* is used only in the first trimester and *EGFL6* and *DLK1* for both first and second trimester placenta analyses (Supplementary Table S4). For endothelial cells, *PECAM1* is the most used marker gene regardless of the GA (Fig. 2). *CDH5*, *PECAM1*, and *CD34* were used to identify only endothelial cells, regardless of placental age (Supplementary Table S4). *MYH11* is the only commonly used gene in second trimester and term placentas for perivascular cells. Interestingly, none of first trimester placental analyses identified perivascular cells. Genes exclusively used to define HCs are similar between the studies regardless of GA, i.e. *CD14*, *CD68*, *CD163*, *MAF*, *CSF1R*, and *AIF1* (Supplementary Table S4). *CD14* is the most used marker to define HCs (Fig. 2) and *CD209* is found only in the term placenta to define HCs (Supplementary Table S4). Nevertheless, subtypes have been only observed for stromal and HCs. Interestingly, no scRNAseq analysis has defined or further characterized subtypes for vascular and perivascular cell types, meaning further research is required.

#### Stromal cells

Han *et al.* (2020) defined three clusters of fetal stromal cells but did not characterize them further. Using first trimester placentas, a proliferative state was identified (Suryawanshi *et al.*, 2018; Shannon *et al.*, 2022), but this subtype was not identified by the only group that identified and fully characterized fetal stromal cell subtypes in the term placenta (Pique-Regi *et al.*, 2019, 2020).

In the first trimester, one common stromal cell subtype expressing *IL6*, a pro-inflammatory gene, was identified in two studies (Suryawanshi *et al.*, 2018; Vento-Tormo *et al.*, 2018). Suryawanshi *et al.* identified two other stromal cell subtypes that both have characteristics of myofibroblasts with up-regulation of extracellular matrix and the smooth-muscle actin-related genes, but differed in the expression of *REN*, *AGTR1*, *IGFBP7*, and *AREG*. Nevertheless, only the subtype with high expression of all these genes was identified by immunofluorescence in the villous stroma (Suryawanshi *et al.*, 2018). Also using first trimester placentas, Liu *et al.* (2018b) observed one subtype expressing *DLK1* and regulating cell adhesion and migration, whilst the other participates in vessel and mesenchymal development.

Using term placentas, Wang *et al.* (2022) identified seven subtypes of stromal cells from both maternal and fetal origin with up-regulation of *DLK1*, *DIO3*, *SOD3*, *TAGLN*, *VEGFA*, *THY1*, and *GDNF*, respectively. Moreover, Pique-Regi *et al.* (2019) revealed that all stromal cells from placental villi presented up-regulation of genes involved in smooth muscle contraction, and apelin and oxytocin signaling pathways, implying the presence of only myofibroblasts, as suggested in first trimester placentas (Suryawanshi *et al.*, 2018). By comparing stromal cells from maternal, internal, and fetal placental sections, Pique-Regi *et al.* (2019) also demonstrated different functions of stromal cells according to their microenvironment. Using second trimester placentas and snRNAseq, the same group did not observe all the previously mentioned subtypes or the same cell proportions (Pique-Regi *et al.*, 2020).

#### Hofbauer cells

Only one scRNAseq study identified two subtypes of HCs (Liu *et al.*, 2018b). After further analysis, one subtype was defined as quiescent and the other one as active as it expressed genes related to HLA class II histocompatibility antigen presentation, cytokine stimuli, inflammatory response, and myeloid leukocyte activation. The authors suggested that this subtype could be involved in the removal of dead cells/debris during development (Liu *et al.*, 2018b).

Despite the identification of all cell types by scRNAseq and snRNAseq, STBs and GCs are under-represented in scRNAseq suggesting that polynucleated cells are not properly identified in these studies and that snRNAseq is probably more appropriate for placental analysis. Furthermore, there are discrepancies between studies regarding the definition of subtypes (Table 2) and markers to define cell types (Supplementary Table S4). It can thus be complicated for biologists who are not specialists in data analysis to know how to choose good markers and compare their cell types with others.

## Meta-analysis of raw datasets: integration of datasets and identification marker genes

### Integration of single-cell RNAseq data

To improve consistency between studies and facilitate future annotation of scRNAseq data of human placentas, we integrated the publicly available raw data and curated gene lists obtained for each cell type. The aim was to create a list with consensus gene markers for fetal cell types in the placenta.

Therefore, we accessed five raw datasets for the first trimester (Vento-Tormo *et al.*, 2018; Saha *et al.*, 2020; Sun *et al.*, 2020; Shannon *et al.*, 2022; Zheng *et al.*, 2022) and three datasets from term placentas (Lu-Culligan *et al.*, 2021; Yang *et al.*, 2021; Garcia-Flores *et al.*, 2022; Zhang *et al.*, 2022; Zhou *et al.*, 2022; Campbell *et al.*, 2023). Poor-quality cells were excluded by Scanpy 1.9.3 (minimum counts = 1000, minimum genes = 500, max genes = 5000, and percentage of mitochondrial genes <15%) (Wolf *et al.*, 2018). Scrublet 0.2.3 was used to remove the doublets (Wolock *et al.*, 2019). Placentas without pathologies in the same trimester were integrated using Scanorama 1.7.3 (Hie *et al.*, 2019). During the integration, Scanorama provided an alignment score for all pairs of datasets by computing the percentage of the cells in each dataset involved in a mutual nearest neighbor matching and taking the maximum of the two percentages for that pair, i.e. the higher alignment score meaning more correlated datasets. Studies with alignment scores <0.15 with all other studies were excluded from the downstream analysis. Integrated datasets were then clustered using Scanpy (15 local neighborhoods for manifold approximation, 30 principal components and resolution of 0.2 for UMAP). Using FindAllMarkers (Seurat v4.3.0) with MAST test, we obtained DEGs (at least expressed in 10% of the cell cluster with fold-change >1.5 and adjusted *P*-value <0.05). As we focused on the fetal-origin cells and maternal immune cells have been well described elsewhere, clusters that expressed *VIM*, *HLA-A*, *HLA-B* and *HLA-C*, *CD45* (also known as *PTRC* and a marker of all leukocytes) and did not express well known markers of HCs were excluded from our analysis.

DEGs from the integrated analysis were compared with our curated marker gene list (Supplementary Table S4) and the marker list obtained from our comparison of annotated datasets (Supplementary Table S5). For trophoblast types from first trimester placentas, DEGs that were observed at the intersection of the integration and at least one of two other lists were selected. As in term placentas, only two studies were included in the comparison of already annotated datasets, and too many DEGs were identified in this analysis. Therefore, we only considered the genes that were common among all analyses, and genes that were previously used as markers (Supplementary Table S4) and DEGs in the integrated analysis.

For both first trimester and term placentae, we then analyzed their expression pattern in the integrated data using dotplot to define markers that were expressed mostly in one cell type, i.e. >0.8 scaled expression and >50% of the cells in the considered cell type (>0.5 for the comparison trophoblast versus non-trophoblast cells and >50% cells in all types for trophoblast versus non-trophoblast cells, no other cell type with >0.8 scaled expression). Finally, we verified their specific expression in the literature using *in situ* hybridization (ISH), immunological staining, spatial transcriptomics, and cell cultures. The genes that met all these criteria were defined as specific marker genes for each trophoblast cell type.

For non-trophoblast cells, as no comparison of already annotated datasets was possible, we selected genes that were observed both in the integration DEG and curated marker lists. Then we also compared the expression of marker genes from first trimester and term analysis to chart their expression between two GAs.

### The first trimester placenta

**Figure 5.**
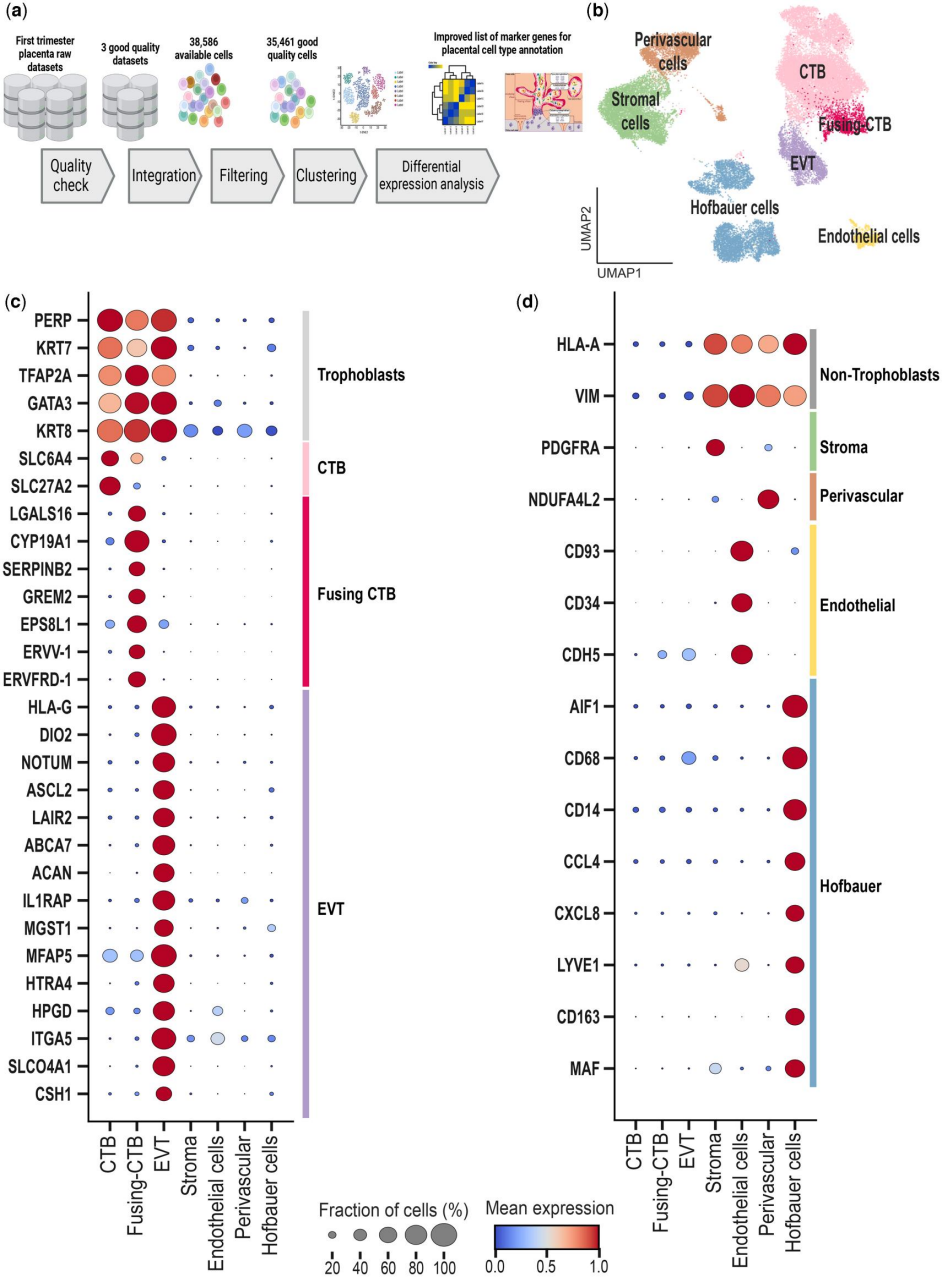
**Results of integration of raw data from first trimester human placentas**. (**a**) Representation of the process for the integration. (**b**) UMAP of the integration. Dotplots of the genes identified using the comparison of already annotated datasets, the curation of markers genes and integration for trophoblasts (**c**), and non-trophoblasts (**d**). CTB: cytotrophoblast; EVT: extravillous trophoblast; UMAP: Uniform Manifold Approximation and Projection.

From the five available datasets, one study deposited an empty data table (Saha *et al.*, 2020) and another study (Zheng *et al.*, 2022) was excluded because of a weak alignment score (<0.12) with the rest of studies (Supplementary Fig. S2a). Using the three remaining datasets (EMTAB6701, GSE131696, and GSE174481, total of 18 individuals), from the 38 586 available cells, 35 461 cells were curated (Fig. 5a). In the first trimester data integration, 482 cells with a good quality were identified as maternal immune cells and were excluded before further analysis.

The integration resulted in seven clusters corresponding to all trophoblast and non-trophoblast cells identified in previous analyses of the first trimester (Fig. 5b). All clusters consisted of cells from the three different datasets (Supplementary Fig. S2b and Supplementary Table S6). From differential expression analysis, DEGs were generated for each cell types (Supplementary Table S7 and Supplementary Fig. S2c and d) and then further analyzed using dot plot scaling (Supplementary Table S8).

#### Trophoblast cells

We identified 150 DEGs between trophoblast and non-trophoblast cells (Supplementary Table S7 and Supplementary Fig. S2c) with only 10 (*PERP*, *KRT7*, *TFAP2A*, *GATA3*, *ERVW-1*, *KRT8*, *EGFR*, *CGA*, *GATA2*, and *TFAP2C*) previously used as markers for trophoblasts in first trimester placenta. Nonetheless, only *PERP*, *KRT7*, *TFAP2A*, *GATA3*, and *KRT8* were shown to be expressed in all trophoblast types but not in non-trophoblast cells using dotplot analysis, confirming that they are good markers of trophoblast types in scRNAseq (Fig. 5c, Supplementary Tables S6 and S7). Therefore, none of these genes appear to specifically define only one trophoblast type as they are expressed in all trophoblast types. Despite the expression in all trophoblast types, differences in expression among the trophoblast types do exist for these genes, explaining why they are often also observed in the DEG lists of the different trophoblast types.

In our integrated dataset, trophoblast cells account for 51.8% of the cells. As previously described, CTBs were the most abundant cell type (39.5% of total cells) and of the trophoblast cells (>77%). Conversely, STBs and EVTs represent <8% of all cells and between 8% and 14% of the trophoblasts (Supplementary Table S6). This agrees with our systematic review of the scRNAseq literature. As all datasets were produced using scRNAseq, it is not surprising to see more cells defined as CTB than STB. As discussed earlier, it is unlikely that the cells previously identified as STB were properly fused STB. For this reason, in our integrated dataset, we decided to name them fusing-CTB.

##### Cytotrophoblasts

For first trimester CTBs, 190 DEGs were identified (Supplementary Table S7 and Supplementary Fig. S2d), among which 13 were previously used as markers (Fig. 2 and Supplementary Table S4). By comparing the 190 DEGs with DEGs from the comparison of annotated datasets, nine genes were common to three of the four studies. Interestingly, none of the 190 DEGs from the integrated analysis were shared with the DEG list for CTBs identified in Suryawanshi *et al.* (2018). Importantly, all the genes that we defined as good markers for all trophoblast types in the previous section appear in the list here. This is not surprising as most of the trophoblasts are CTBs but also suggests that these genes are not specific to one trophoblast subtype, despite high expression in CTB cells.

Altogether, 20 genes were identified by at least the integration and the comparison of already annotated datasets or curated marker genes analyses. Among these, *DUSP9* and *SLC22A1* were identified in the three analyses; *GSTA3*, *MSX2*, *SEMA6A*, *SLC13A4*, *SLC52A1*, *SLC6A4*, and *SIGLEC6* were identified in the integration and the annotated dataset comparison; *SMAGP*, *BCAM*, *VGLL1*, *SLC27A2*, *XAGE3*, *PEG10*, *PAGE4*, *ISYNA1*, *EGFR*, *CCR7*, and *PARP1* were identified in the integration and the curated marker lists. However, only *SLC6A4* and *SLC27A2* were specific markers of CTBs in the first trimester (Fig. 5c and Supplementary Table S8). All the other genes were either expressed at the same level in fusing-CTBs and/or EVTs or with a higher expression in fusing-CTBs. This demonstrates the difficulty of distinguishing CTBs from fusing-CTB using scRNAseq data in the first trimester placentas.

In both primary human trophoblasts from term placentas and BeWo cells, *SLC6A4* (also known as *SERT*) was expressed more in STBs than in CTBs (Vachalova *et al.*, 2022). Furthermore, *SLC6A4* was also identified in STBs of term placentas using ISH (Bottalico *et al.*, 2004). In human primary trophoblasts from term placentas, expression of *SLC27A2* was significantly higher after syncytialization (Kallol *et al.*, 2018) and was also expressed more in STBs than CTBs in second trimester placentas (Gormley *et al.*, 2021). No literature regarding their expression in the first trimester placentas could be recovered. Therefore, it is likely that *SLC6A4* and *SLC27A2* are good markers for CTB using scRNAseq but only in the first trimester placentas.

Using spatial transcriptomics and snRNAseq, Arutyunyan *et al.* (2023) demonstrated that *BCAM* was only expressed in CTBs. In our integrated analysis, around 60% of the fusing-CTB expressed *BCAM* at a lower level than CTBs. This gene could probably also be considered as a good marker of CTB using scRNAseq. Furthermore, as it is expressed in >50% of the EVT in the integrated dataset, *PARP1* was not selected but this expression is very low (<0.02), suggesting that *PARP1* could also be a good marker for CTB in scRNAseq. This seems to be confirmed by the literature, as in healthy term placentas, *PARP1*, is specifically expressed by CTB (Longtine *et al.*, 2012).

Taken together, only *BCAM*, *SLC6A4*, *SLC27A2*, and likely *PARP1* can be used as specific markers of CTB in the first trimester placentas using scRNAseq.

##### Fusing-CTB

In fusing-CTBs from the first trimester, 226 DEGs were identified (Supplementary Table S7 and Supplementary Fig. S2d), among which 16 were previously used as marker for STBs in the first trimester placentas (Fig. 2 and Supplementary Table S4). When comparing the 226 DEGs with our annotated dataset comparison, 18 genes were common in at least three of the four studies. Altogether 34 genes were identified in the integration analysis and annotated datasets or curated marker genes analyses: *LGALS16*, *SERPINB2*, *GREM2*, *EPS8L1*, *ERVV-1*, *LGALS13*, *KMO*, *GLDN*, *IGSF5*, *ADHFE1*, *PTPN21*, *HES2*, *NEURL1*, *PHYHIPL*, *HSD11B2*, *ENDOU*, *TCL1B*, and *LGALS14* were identified both in the integration and the annotated datasets; and *CYP19A1*, *GDF15*, *ERVFRD-1*, *INSL4*, *CGA*, *SLC52A1*, *SLC13A4*, *ERVW-1*, *HSD3B1*, *SLC22A11*, *TFAP2A*, *LCMT1-AS2*, *PAGE4*, *KISS1*, *PSG6*, and *PSG2* were identified in the integration and the curated marker lists.

Interestingly, in contrast to the CTB, most of the genes identified in at least two analyses were mainly expressed in fusing-CTBs but only *LGALS16*, *CYP19A1*, *SERPINB2*, *GREM2*, *EPS8L1*, *ERVV-1*, and *ERVFRD-1* were expressed in >50% of fusing-CTB (Supplementary Table S8).

Several studies using different methods (immunohistochemistry, immunofluorescence, and ISH) demonstrated that galectin family genes, such as *LGALS16*, are expressed in STB but not in CTB (Than *et al.*, 2004, 2014; Huppertz *et al.*, 2008). *CYP19A1* (encoding the aromatase cytochrome P450) was exclusively observed in STB compared to underlying CTB and villous core using immunohistochemistry (Fournet-Dulguerov *et al.*, 1987). *SERPINB2* protein was observed in the cytoplasm of STB but not in CTB or EVT (Feinberg *et al.*, 1989). Similarly, *GREM2* expression coincides with the activation of STB genes and hCG secretion (Sudheer *et al.*, 2012). *ERVV-1* was also highly expressed by differentiated trophoblast cultures compared to undifferentiated (Renaud *et al.*, 2015). Using human trophoblast stem cells (hTSCs) and BeWo cells, *ERVFRD-1* was shown to be nearly absent before differentiation and significantly upregulated after differentiation in STB (Renaud *et al.*, 2015; West *et al.*, 2022), suggesting that it is indeed a specific marker of STB that can be present in the early stages of the fusion process. No further information could be found to validate the use of *EPS8L1* as marker of fusing-CTB in human placenta.

Using spatial transcriptomics and snRNAseq, Arutyunyan *et al.* (2023) also demonstrated that *ERVV-1*, *ERVFRD-1*, and *GREM2* were markers of fusing-CTB and not STB, supporting the immature state of STB in the integrated analysis. *ERVV-1*, *GLDN*, *LGALS13*, and *LGALS16* were also identified by HuBMAP and/or Cellmarker databases as STB markers. In the Descartes cell atlas (based on data from Cao *et al.*, 2020), *LGALS13*, *LGALS16*, and *ADHFE1* were associated with ‘trophoblast giant cells’ that were defined with pregnancy-specific glycoprotein (PSG) genes (*PSG4*, *PSG6*, and *PSG9*). *LGALS13*, *LGALS16*, and *GDF15* were also used to define EVT or CTB in at least one of these databases.

Therefore, we concluded that *LGALS16*, *SEPRINB2*, *CYP19A1*, *GREM2*, *ERVV-1*, and *ERVFRD-1* can be used as specific markers of fusing-CTB in the first trimester placentas.

##### Extravillous trophoblast

Altogether, 228 DEGs were identified in EVT (Supplementary Table S7 and Supplementary Fig. S2d), among which 19 genes were previously used as markers for EVT in the first trimester placentas (Fig. 2 and Supplementary Table S4). When comparing all DEGs with the gene list identified from annotated datasets, 10 genes were found in at least three of the four studies. Altogether, 29 genes were identified by at least two analyses, i.e. *ASCL2*, *ABCA7*, *ACAN*, *IL1RAP*, *MGST1*, *KRT14*, *KRT17*, *KISS1R*, *FAT2*, and *SAA1* were identified in the integrated analysis and the list of annotated datasets; and *HLA-G*, *DIO2*, *NOTUM*, *LAIR2*, *MFAP5*, *HTRA4*, *HPGD*, *ITGA5*, *SLCO4A1*, *CSH1*, *HSD3B1*, *MMP2*, *CGA*, *FN1*, *MMP12*, *FSTL1*, *HLA-C*, *PAPPA2*, and *PRG2* were identified in the integrated analysis and the curated marker list.

In the integrated data, *HLA-G*, *DIO2*, *NOTUM*, *ASCL2*, *LAIR2*, *ABCA7*, *ACAN*, *IL1RAP*, *MGST1*, *MFAP5*, *HTRA4*, *HPGD*, *ITGA5*, *SLCO4A1*, and *CSH1* were specifically expressed in EVT (Supplementary Table S8).

*HLA-G* is the most used EVT signature gene (Supplementary Table S4) and is well known to be expressed only in EVT compared to other trophoblasts (McMaster *et al.*, 1995; Hackmon *et al.*, 2017b). In the first trimester placentas, *DIO2* was expressed in CTB, STB, and CCC by immunofluorescence (Adu-Gyamfi *et al.*, 2021). *NOTUM* was more expressed in EVT compared to CTB differentiated from hTSC (Jeyarajah *et al.*, 2022) but was observed in all trophoblasts in the second trimester placentas by immunohistochemistry (Robinson *et al.*, 2017). *ASCL2* is a specific transcription factor for the EVT lineage with expression in the EVT columns in human 12 week-old placentas, and its deficiency results in increased STBs and reduced EVTs differentiation (Varberg *et al.*, 2021, 2023). In addition, *ASCL2* expression is decreased across differentiation in EVT and almost absent once the progenitor state is lost (Renaud *et al.*, 2015). Using ISH and immunohistochemistry, *LAIR2* was shown to be expressed only in the more distal portions of CCC and the invading EVT in the first trimester placentas, while no expression was observed in any other fetal cells (Founds *et al.*, 2010). From the microarray profiling of CTB and EVT extracted from the first trimester placentas, *ACAN* was shown to be overexpressed in EVT compared to CTB (Oravecz *et al.*, 2022). In contrast, *HTRA4* expression was shown to increase during syncytialization of BeWo cells (Mansilla *et al.*, 2021) and in term placentas, *HTRA4* protein localizes mainly to STB using immunohistochemistry (Liu *et al.*, 2018a). *HPGD* was shown to be primarily expressed by EVT, with a weak expression in STB in the first trimester and term placentas (de Assis *et al.*, 2022). Using PCR array on CTB and EVT isolated from the first trimester placentas, *ITGA5* was shown to be upregulated in EVT compared to CTB (DaSilva-Arnold *et al.*, 2015). *SLCO4A1* was expressed by both CTB and STB after differentiation from primary tissue of first trimester placentas (Berveiller *et al.*, 2015). *CSH1* was shown to be expressed both in STB and EVT from placental *in vitro* models (Takao *et al.*, 2011). No further information could be found to validate the use of *ABCA7*, *IL1RAP*, *MGST1*, and *MFAP5* as markers of EVT in human placenta.

In conclusion, *HLA-G*, *NOTUM*, *ASCL2*, *LAIR2*, *ACAN*, *HPGD*, and *ITGA5* were shown to be good markers for EVT of the first trimester placentas.

#### Non-trophoblast cells

Altogether, 302 DEGs were related to non-trophoblast cells (Supplementary Table S7 and Supplementary Fig. S2c) with four (*HLA-A*, *HLA-B*, *HLA-C*, and *VIM*) previously used to define them in first trimester placentas. *HLA-B* presented a scaled expression <0.5 in endothelial cells and *HLA-C* was highly expressed in EVT (Fig. 5d and Supplementary Table S8). Therefore, only *HLA-A* and *VIM* met the criteria to be specific markers of non-trophoblasts. Owing to this shared expression between non-trophoblast cell types, none of these genes appeared to be specific markers of only one non-trophoblast type.

In the integrated data, stromal cells are the second largest cluster (19% of total placental cells) and the most abundant (40%) among the non-trophoblast cells followed by HCs (30%) (Supplementary Table S6). Unlike other studies on first trimester placentas, our integrated analysis also identified perivascular cells in addition to stromal, endothelial, and HCs.

##### Stromal and perivascular cells

Altogether, 392 DEGs were identified (Supplementary Table S7 and Supplementary Fig. S2d) with 15 genes previously used as markers for stromal cells in the first trimester placentas: *COL1A1*, *PITX2*, *COL6A2*, *COL3A1*, *COL1A2*, *EGFL6*, *DLK1*, *PDGFRA*, *ACTA2*, *ANGPTL2*, *VIM*, *CTGF*, *ANGPTL1*, *THY1*, and *ANGPTL4* (Fig. 2 and Supplementary Table S4). However, most of them (i.e. *EGFL6*, *COL1A1*, *COL1A2*, *COL3A1*, *COL6A2*, and *DLK1*) were also expressed in the perivascular cells, demonstrating the difficulties of identifying them in the first trimester placentas (Supplementary Table S8). Only *PDGFRA* was not expressed in perivascular cells and, therefore, met all criteria to be a specific marker of stromal cells in the first trimester placentas.

In perivascular cells, 329 DEGs were identified (Supplementary Table S7 and Fig. 2d), with *NDUFA4L2*, *ACTA2*, and *TAGLN* being used to identify perivascular cells in term placentas (no available marker from first trimester studies as perivascular cells were not defined). After this analysis, *NDUFA4L2* is the only good marker to define perivascular cells (Supplementary Table S8).

##### Endothelial cells

Altogether, 278 DEGs were identified (Supplementary Table S7 and Supplementary Fig. S2d) with nine genes previously used as markers for endothelial cells in the first trimester placentas: *CD93*, *CD34*, *VIM*, ITGA*2*, *CDH5*, *KIT*, *NRN1*, *ZEB1*, and *PECAM1* (Supplementary Fig. S2 and Supplementary Table S4). Except for *VIM* and *NRN1*, these genes were specifically expressed in endothelial cells (Supplementary Table S8). However, only *CD93*, *CD34*, and *CDH5* were expressed in >50% of endothelial cells, allowing them to be considered as good markers of this cell type in first trimester (Fig. 5d and Supplementary Table S7).

##### Hofbauer cells

Altogether, 220 DEGs were identified (Supplementary Table S6 and Supplementary Fig. S2d) with 11 genes previously used as markers for HCs in the first trimester placentas: *AIF1*, *CD68*, *CD14*, *CCL4*, *CXCL8*, *LYVE1*, *CSF1R*, *CD163*, *POU2F2*, *MAF*, and *PTPRC* (Supplementary Fig. S2 and Supplementary Table S4). *AIF1*, *CD68*, *CD14*, *CCL4*, *CXCL8*, *LYVE1*, *CD163*, and *MAF* met the criteria (Supplementary Table S8), allowing them to be considered as specific markers of HCs in first trimester placentas.

### Term placentas

**Figure 6.**
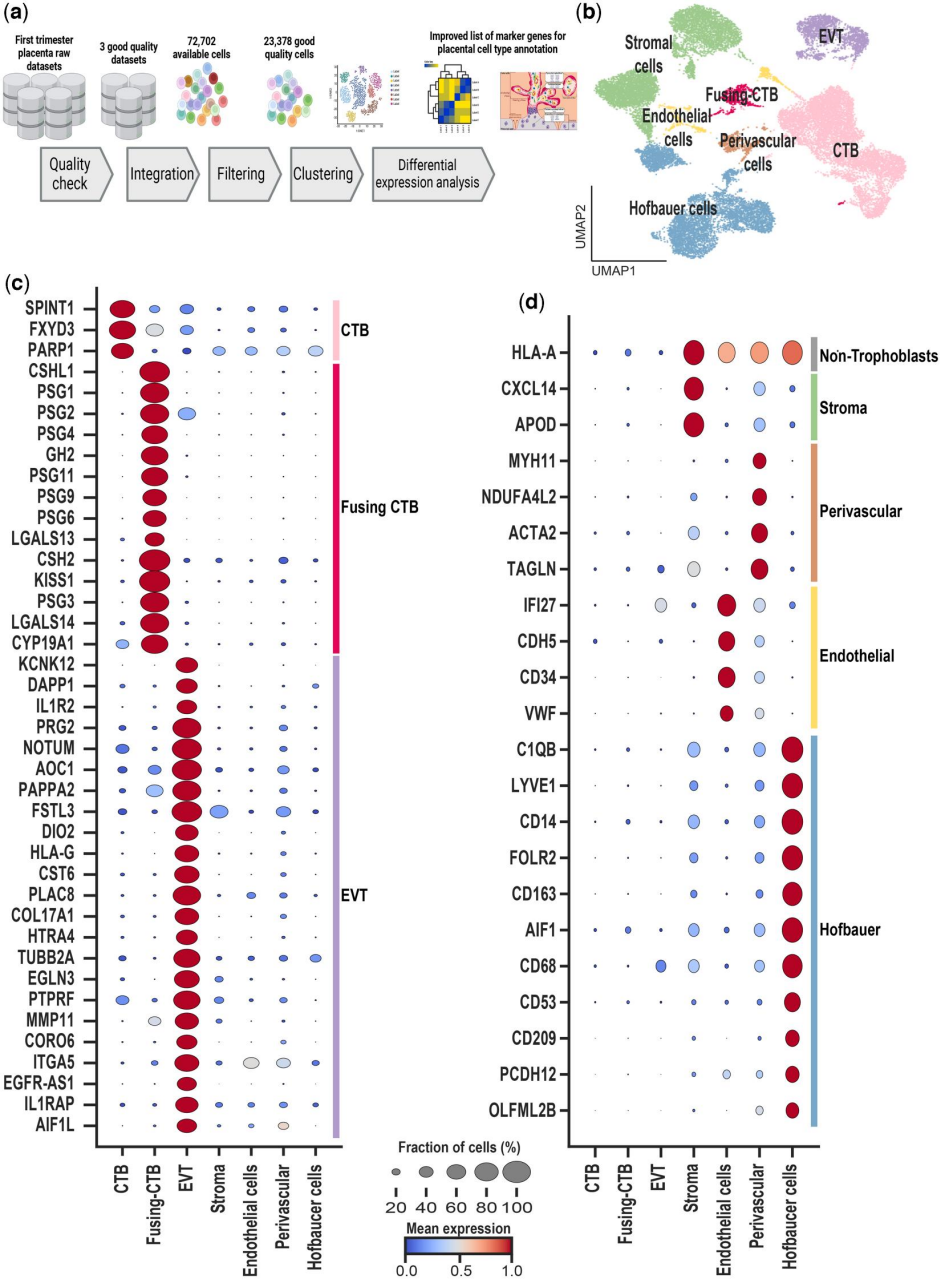
**Results of integration of raw data from term human placentas**. (**a**) Representation of the process for the integration. (**b**) UMAP of the integration. Dotplots of the genes identified using the comparison of already annotated datasets, the curation of markers genes, and integration for trophoblasts (**c**) and non-trophoblasts (**d**). CTB: cytotrophoblast; EVT: extravillous trophoblast; UMAP: Uniform Manifold Approximation and Projection.

Only three raw datasets were available for integration (Lu-Culligan *et al.*, 2021; Yang *et al.*, 2021; Zhang *et al.*, 2021a; Zhou *et al.*, 2022; Campbell *et al.*, 2023). All datasets presented alignment score (>0.15) with all other studies (Supplementary Fig. S3a). From the three datasets (GSE182381, GSE171381, and GSE173193, which included nine individuals), 23 378 cells remained after filtering 72 702 available cells (i.e. 25 972 cells failed the quality control and 23 352 were maternal immune cells) (Fig. 6a and Supplementary Table S6). Unlike the first trimester placentas, half of the cells in the available raw datasets were maternal immune cells. As human placenta bathes in maternal blood after the first trimester, such a high quantity of maternal immune cells likely comes from maternal immune cell infiltration into term placentas. Therefore, only 571 cells were used for the integration analysis from the total 4368 cells that passed the quality control in Campbell *et al.* (2023) (Supplementary Table S6).

The same seven clusters of cell types as those in the first trimester were identified in term placentas, including all trophoblast and non-trophoblast cells identified in previous scRNAseq analyses (Fig. 6b). Cells from the three datasets consisted of all clusters (Supplementary Fig. S3b and Supplementary Table S6). We first determined the top 10 DEGs for each cell type (Supplementary Fig. S3) and then cross-compared the genes with the annotated datasets and curated marker lists (Fig. 6 and Supplementary Fig. S2).

#### Trophoblast cells

Altogether, 313 DEGs were obtained in trophoblasts versus non-trophoblast cells (Supplementary Table S8) with four genes already used as markers of trophoblast cells in term placentas: *KRT8*, *KRT7*, *PERP*, and *CYP19A1*. Only *KRT8* was expressed in all trophoblast types but it was also expressed in 50% of the HCs. Therefore, none of them met the criteria to be specific markers.

Trophoblasts represented only 43.2% of the cells, almost 10% less than those in the first trimester placentas. As for the first trimester, CTBs were the most abundant type (32.6% of total cells and more than 75% of trophoblast cells), while STBs and EVTs also represent <10% of all cells and, respectively, 5.8% and 18.7% of the trophoblasts (Supplementary Table S6). Similar to the first trimester, it is unlikely that the cells identified as STB in scRNAseq were properly fused STB. Accordingly, we decided to name them fusing-CTB in this meta-analysis.

##### Cytotrophoblast

Altogether, 307 DEGs were identified (Supplementary Table S9) with 10 genes being identified by the three analyses (the integration, comparison of already annotated datasets, and curated marker genes): *PAGE4*, *PHLDA2*, *IFI6*, *TINAGL1*, *SPINT1*, *FXYD3*, *MGST3*, *KRT18*, *PARP1*, and *SLC27A2*. Furthermore, five genes (*INSL4*, *EGFR*, *KRT7*, *GATA3*, and *CDH1*) previously identified in the curated marker list were in the DEG list of the integrated analysis.

As for the first trimester analysis, most of the genes here were also expressed in fusing-CTB (Supplementary Table S8). However, *SPINT1*, *FXYD3*, *MGST3*, *KRT18*, and *PARP1* met all the criteria (scaled expression in CTB >0.8, no other cell type with scaled expression >0.8, expression in at least 50% of CTB) and could be considered as specific markers of CTB.

Using immunohistochemistry, *SPINT1* was increased throughout the pregnancy in CTBs with a clear specific staining in CTBs of term placentas (Mori *et al.*, 2007). As for the first trimester placentas, *PARP1* was specifically expressed by CTB in healthy term placentas. However, it was also expressed in STBs in damaged regions in the placentas of pre-eclampsia or intra-uterine growth restriction (Longtine *et al.*, 2012). The expression of *KRT18* was detected in all trophoblasts differentiated from human KRT7+ induced placentas stem cells with bone morphogenetic protein 4 treatment (Tsuchida *et al.*, 2020). No further information could be found to validate *FXYD3* and *MGST3* as marker of CTB in human term placentas. Taken together, only *SPINT1* was confirmed as a specific marker of CTBs in term placentas.

##### Fusing-CTB

For fusing-CTBs, 119 DEGs were identified (Supplementary Table S8) with six genes (*CSH2*, *KISS1*, *PSG3*, *LGALS14*, *CGA*, and *CYP19A1*) identified by the three analyses. Furthermore, 12 genes were common in the curated marker list and the integrated analysis: *CSHL1*, *PSG1*, *PSG2*, *PSG5*, *PSG4*, *GH2*, *PSG11*, *PSG9*, *PSG6*, *LGALS13*, *HPGD*, and *SEMA3B*. Most of these genes (*CSHL1*, *PSG1*, *PSG2*, *PSG4*, *GH2*, *PSG11*, *PSG9*, *PSG6*, *LGALS13*, *CSH2*, *KISS1*, *PSG3*, *LGALS14*, and *CYP19A1*) were identified as specific markers in term placentas.

*CSHL1*, *CSH2*, and *GH2* exhibited almost 30, 40, and 10 times higher expression in STB, respectively, compared to those in the undifferentiated primary CTBs in healthy term placentas (Jiang *et al.*, 2019). *PSG1*, *PSG3*, and *PSG9* transcripts increased after differentiation into STBs from both JEG-3 cells and primary CTBs in term placentas (Camolotto *et al.*, 2010). Furthermore, using one monoclonal antibody specific to PSG proteins, their specific expression in STBs was confirmed in both first trimester and term placentas using immunohistochemistry (Zhou *et al.*, 1997). Therefore, all PSGs can probably be considered as good markers of fusing-CTB. Furthermore, hTSCs with *MSX2* depletion led to elevated *CSH2* and *CYP19A1* expression owing to differentiation into STBs (Hornbachner *et al.*, 2021). *CYP19A1* was exclusively observed in STBs compared to the underlying CTBs and villous core using immunochemistry (Fournet-Dulguerov *et al.*, 1987). In the first trimester placentas, *KISS1* expression was specific in STBs using both ISH and immunohistochemistry (Bilban *et al.*, 2004). However, more recent studies demonstrated that CTBs also expressed *KISS1* (Park *et al.*, 2011; Wu *et al.*, 2014). Various methods (immunohistochemistry, immunofluorescence, and ISH) were used to demonstrate that galectin family genes (*LGALS13*, *LGALS14*, and *LGALS16*) were expressed in STBs but not in CTBs (Than *et al.*, 2004, 2014; Huppertz *et al.*, 2008). However, quantitative PCR (qPCR) assay indicated that EVTs also expressed *LGALS13*, although the expression was lower compared to STB (Ogushi *et al.*, 2023).

Therefore, we concluded that *CSHL1*, *PSG1*, *PSG2*, *PSG4*, *GH2*, *PSG11*, *PSG9*, *PSG6*, *CSH2*, *PSG3*, *LGALS14*, and *CYP19A1* can be used as specific markers of fusing-CTB in term placentas.

##### Extravillous trophoblast

Altogether, 222 DEGs were identified in EVTs (Supplementary Table S9) with 31 genes observed by the three analyses: *FN1*, *PRG2*, *NOTUM*, *AOC1*, *PAPPA2*, *TNFSF10*, *HPGD*, *FLT1*, *FSTL3*, *EBI3*, *KRT19*, *DIO2*, *HLA-G*, *HTRA1*, *TPM1*, *CST6*, *PLAC8*, *KRT7*, *COL17A1*, *HTRA4*, *TUBB2A*, *EGLN3*, *PTPRF*, *MMP11*, *MCAM*, *CORO6*, *ITGA5*, *EGFR-AS1*, *IL1RAP*, *ENG*, and *AIF1L*. All these genes were highly expressed in EVT in term placentas but only *KCNK12*, *DAPP1*, *IL1R2*, *PRG2*, *NOTUM*, *AOC1*, *PAPPA2*, *FSTL3*, *DIO2*, *HLA-G*, *CST6*, *PLAC8*, *COL17A1*, *HTRA4*, *TUBB2A*, *EGLN3*, *PTPRF*, *MMP11*, *CORO6*, *ITGA5*, *EGFR-AS1*, *IL1RAP*, and *AIF1L* were specifically expressed in EVTs. Furthermore, five genes, i.e. *TAC3*, *KCNK12*, *DAPP1*, *IL1R2*, and *PROCR*, were also identified as curated markers from the integrated analysis.

*KCNK12* and *ITGA5* expression were higher in human primary placental EVTs than CTBs from the first and second trimester (Apps *et al.*, 2011; Telugu *et al.*, 2013). *IL1R2* was demonstrated to be expressed by a small population of primary CTBs and HTR8/SVneo cells. However, once differentiated into STB or EVT, these cells no longer expressed *IL1R2* (Takao *et al.*, 2011). *PRG2* was detected only in EVTs that invade venous and lymphatic vessels in first trimester placentas by immunofluorescence. In cell culture assays, *PRG2* was observed only in differentiated EVTs and not in differentiated STBs from primary CTBs from first trimester placentas (Windsperger *et al.*, 2017). Similarly, *NOTUM* was expressed by all trophoblast types in the second trimester placentas by immunohistochemistry (Robinson *et al.*, 2017). *AOC1*, which encodes the D-amino acid oxidase protein, was demonstrated by immunofluorescence to be expressed only in invasive EVTs and was only secreted by primary EVTs from first trimester placentas compared to other trophoblasts (Velicky *et al.*, 2018; Haider *et al.*, 2022). *PAPPA2* appeared to be controversial as its expression was reported in all trophoblasts (Windsperger *et al.*, 2017) and only in invasive EVTs in both first and term placentas (Haider *et al.*, 2022). *FSTL3* was recently found in all trophoblast types, regardless of GA (Wu *et al.*, 2023). *DIO2* was expressed in CTBs, STBs, and CCCs by immunofluorescence (Adu-Gyamfi *et al.*, 2021), but no information is available for term placentas. Expression of *HLA-G* in EVTs was detected with qPCR, immunohistochemistry, and western blot throughout pregnancy (Hackmon *et al.*, 2017a). *HLA-G* was also the most used EVT signature gene in term placentas (Fig. 2 and Supplementary Table S4) and was expressed only in EVTs compared to other trophoblasts (McMaster *et al.*, 1995; Hackmon *et al.*, 2017b). However, *CST6* was expressed in other trophoblast types, especially in differentiated STBs. *PTPRF* was decreased once primary CTBs were differentiated into STBs in healthy term placentas (Jiang *et al.*, 2019). *PLAC8* was detected in CCCs and EVTs throughout the pregnancy and specifically in EVTs in term placentas by immunofluorescence and ISH (Chang *et al.*, 2018). Similarly, *COL17A1* was expressed mainly by early ITGA2^+^ CCCs and in some EVTs (Lee *et al.*, 2018). In contrast, *HTRA4* expression increased during syncytialization of BeWo cells (Mansilla *et al.*, 2021) and *HTRA4* protein localized mainly in STB using immunohistochemistry in term placentas (Liu *et al.*, 2018a). Also using immunochemistry, *MMP11* was expressed in decidual stromal cells and EVTs in first trimester placentas (Anacker *et al.*, 2011) but information on term placenta is missing.

No further information could be found to validate the use of *DAPP1*, *TUBB2A*, *EGLN3*, *CORO6*, *EGFR-AS1*, *IL1RAP*, and *AIF1L* as markers of EVT in human placenta. In conclusion, *KCNK12*, *PRG2*, *NOTUM*, *AOC1*, *HLA-G*, *PLAC8*, *COL17A1*, and *ITGA5* could be reliable markers for EVT in term placentas.

#### Non-trophoblast cells

A total of 434 DEGs were related to non-trophoblast cells, with only *HLA-A* previously used as a marker in term placentas. *HLA-A* met all the criteria and can, therefore, be considered as a specific marker for non-trophoblasts in term placentas.

As for the first trimester, stromal cells were the second largest cluster, accounting for 27.8% of total placental cells, and were the most abundant (49%) among the non-trophoblast cells followed by HCs (43.6%) (Supplementary Table S5).

##### Stromal and perivascular cells

In total, 280 DEGs were identified (Supplementary Table S9 and Fig. 6) with 25 genes previously used as markers for stromal cells in term placentas: *DCN*, *COL3A1*, *IGFBP3*, *COL1A1*, *HGF*, *CXCL14*, *APOD*, *COL6A2*, *DLK1*, *VCAN*, *SPARC*, *CALD1*, *MYL9*, *SERPINF1*, *TGFBI*, *TXNIP*, *SOD3*, *TAGLN*, *IFITM3*, *LGALS1*, *ECM1*, *NNMT*, *ACTA2*, *FMOD*, and *PDGFRB* (Fig. 2 and Supplementary Table S4). Like the first trimester placentas, most of these genes were also expressed in perivascular cells. Only *PDGFRB*, *CXCL14*, and *APOD* were specific to stromal cells and, therefore, met all criteria to be specific markers of stromal cells in term placentas.

In perivascular cells, 146 DEGs were identified (Supplementary Table S9 and Fig. 6) with *MYH11*, *NDUFA4L2*, *ACTA2*, and *TAGLN* being previously used. Here, all of them were specific markers of perivascular cells (Supplementary Table S8) and were, therefore, reliable markers to define them.

##### Endothelial cells

Altogether, 227 DEGs were identified in endothelial cells (Supplementary Table S9 and Fig. 6) with six genes previously used as markers in term placentas: *PECAM1*, *IFI27*, *CDH5*, *CD34*, *VWF*, and *PLVAP* (Supplementary Fig. S2 and Supplementary Table S4). Except for *PECAM1* and *PLVAP*, all these genes met the criteria to be specific markers (Supplementary Table S9 and Fig. 6).

##### Hofbauer cells

Altogether, 342 DEGs were identified (Supplementary Table S9, Fig. 6) with only 17 genes previously used as markers for HCs in term placentas: *C1QB*, *LYVE1*, *RNASE1*, *CD14*, *PLTP*, *LGMN*, *FOLR2*, *CD163*, *AIF1*, *CSF1R*, *MAF*, *CD68*, *NPC2*, *CD53*, *CD209*, *PCDH12*, and *OLFML2B* (Supplementary Fig. S2, Supplementary Table S4). All these genes were highly expressed but only *C1QB*, *LYVE1*, *CD14*, *FOLR2*, *CD163*, *AIF1*, *CD68*, *CD53*, *CD209*, *PCDH12*, and *OLFML2B* were not expressed in other cell types and were thus specific markers of HCs (Supplementary Table S9, Fig. 6).

### Dynamics of marker gene expression across gestation

**Figure 7.**
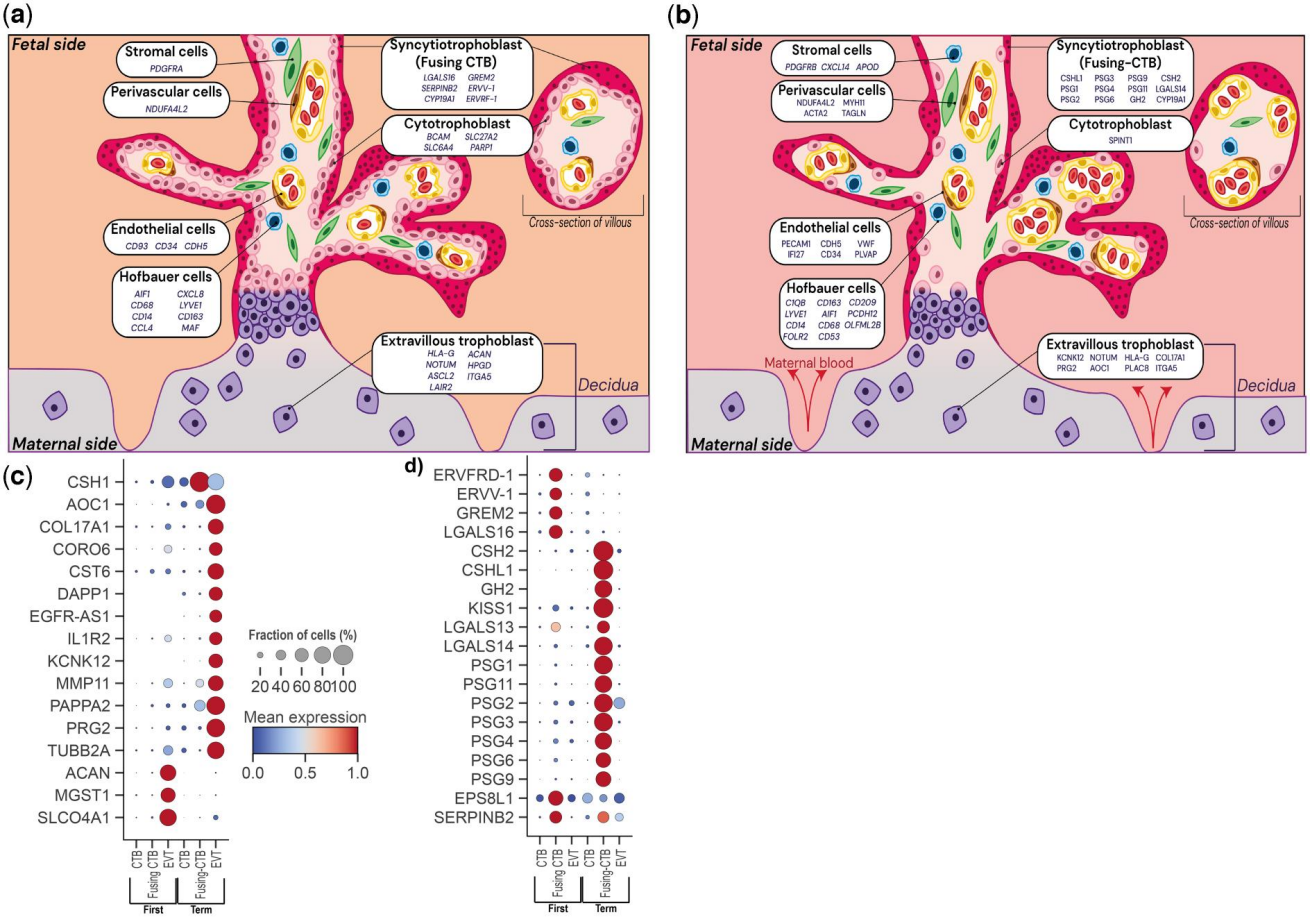
**Specific markers of human first trimester and term placental villous cells and their evolution across pregnancy**. (**a**) Representation of human placenta from first trimester with specific markers identified in this review. (**b**) Representation of human placenta at term with specific markers identified in this review. (**c**) Dotplot of specific markers that particularly highlight the maturation of extravillous trophoblast. (**d**) Dotplot of specific markers that particularly highlight the maturation of syncytiotrophoblasts. CTB: cytotrophoblast.

Our meta-analysis provided a list of specific marker genes for each placental cell type in the first trimester and at term (Fig. 7). Many genes in several cell types, however, did not overlap between the two GA, preventing them to be used as markers for both (Supplementary Table S10).

None of the markers of all trophoblasts were usable in term placentas and only *HLA-A* for non-trophoblasts could be used regardless of the GA (Supplementary Table S10 and Fig. 7). No CTB marker and only *CYP19A1* for STB could be used at both timepoints, while for EVT, *ABCA7*, *ASCL2*, *DIO2*, *EGLN3*, *FSTL3*, *HLA-G*, *HTRA4*, *IL1RAP*, *ITGA5*, *LAIR2*, *NOTUM*, *PLAC8*, and *PTPRF* were specifically expressed at both timepoints (Supplementary Table S10 and Fig. 7). In non-trophoblasts, *CXCL14* and *PDGFRA* for stroma and *NDUFA4L2* for perivascular cells were specifically expressed in both timepoints. Endothelial markers *CD34* and *CDH5* and most of the HC markers (*AIF1*, *C1QB*, *CCL4*, *CD14*, *CD163*, *CD53*, *CD68*, *CXCL8*, and *FOLR2*) were also specific at both timepoints. These results highlight the dynamic gene expression across gestation.

Interestingly, in EVT most of the genes (*AOC1*, *COL17A1*, *CORO6*, *CST6*, *DAPP1*, *EGFR-AS1*, *IL1R2*, *KCNK12*, *MMP11*, *PAPPA2*, *PRG2*, and *TUBB2A*) with a different expression profile between first trimester and term presented a low expression and were expressed by <50% of the EVTs in first trimester placentas (Fig. 7). Moreover, *ACAN*, *MGST1*, and *SLCO4A1* presented the opposite profile with low expression and by <50% of the EVT in term placentas. These genes could therefore represent markers of EVT maturation across gestation.

Similarly, *CSH2*, *CSHL1*, *GH2*, *KISS1*, *LGALS13*, *LGALS14*, *PSG1*, *PSG11*, *PSG2*, *PSG3*, *PSG4*, *PSG6*, and *PSG9* showed low expression and were expressed in <50% of fusing-CTBs in the first trimester placentas, demonstrating the progression to a secretory phenotype in term placentas. Furthermore, fusing-CTB in term placentas did not express *ERVFRD-1*, *ERVV-1*, *GREM2*, and *LGALS16* but around 15% of CTB did. These results suggested that fusing-CTB defined in the first trimester clustered with CTB in term placentas, and that more mature STBs were produced in term placentas by scRNAseq. Despite the more mature phenotype, owing to the dissociation method and the placental structure, it is unlikely that these cells in term placentas are completely fused STB, and more research is needed to confirm this.

Furthermore, *CSH1* was shown to be a specific marker of EVT in the first trimester data (Fig. 5). However, even if more than 50% of EVT still expressed this marker at term, the expression was low (Fig. 7). On the contrary, all the fusing-CTB in term placentas expressed this gene at a very high level indicating that placental lactogen is mainly produced by EVT in the first trimester but by STB at term (Fig. 7). These comparisons demonstrated that trophoblast cells may particularly evolve throughout gestation, highlighting the importance of adapting the specific markers to GA when annotating cell types in a scRNAseq study.

## Discussion

### Contribution of single-cell RNAseq of the maternal–fetal interface to pathologies

To date, placentas from women with recurrent pregnancy loss (RPL), pre-term birth, advanced maternal age, pre-eclampsia, gestational diabetes mellitus (GDM), or severe acute respiratory syndrome coronavirus 2 (SARS-CoV-2) infection have all been compared to healthy placentas using scRNAseq (Table 1).

#### Recurrent pregnancy loss

RPL has been studied with scRNAseq analysis primarily using decidual tissue (Saha *et al.*, 2020; Chen *et al.*, 2021; Du *et al.*, 2021; Guo *et al.*, 2021; Wang *et al.*, 2021). The first study focused on the expression of the *TEAD4* pathway in progenitor CTBs in two normal 6- to 8-week placentas and showed a strong reduction of *TEAD4* expression in villous CTBs and CCCs in RPL placentas, confirming the essential role of *TEAD4* in the establishment of pregnancy and its likely involvement in RPL (Saha *et al.*, 2020). Interestingly, all studies that focused on decidual immune cells have demonstrated that a subset of natural killer cells supporting fetal growth is dramatically diminished, while one other subset with a cytotoxic and immune-active signature is increased in RPL (Chen *et al.*, 2021; Guo *et al.*, 2021; Wang *et al.*, 2021), showing an alteration of the placental immune microenvironment in RPL, which provides clues for future treatments. The only study considering all cell types in the decidua observed aberrant decidualization with alteration of decidual cell subtype proportions, and obstructed communication of decidual cells with other cells, possibly linked to the observed over-activation of the tumor necrosis factor superfamily of genes (Du *et al.*, 2021).

#### Gestational age

Only one study has investigated the effect of GA on placenta and compared preterm (33–35 weeks of gestation) and term placentas (38–40 weeks of gestation) collected with and without labor (n = 3/group). The greatest numbers of DEGs were found in CTBs, EVTs, and maternal macrophages. Furthermore, bulk RNA sequencing of maternal blood at different gestational stages enabled the identification of non-invasive markers for monitoring preterm labor risk (Pique-Regi *et al.*, 2019).

#### Maternal aging

Analysis of three term placentas collected from women >35 years of age versus those from younger mothers showed no changes in cell type distribution. However, EVT function, especially in one subtype playing a role in cell invasion, was affected by maternal age. Extracellular matrix receptor interaction and cell invasion-related genes were also altered in placentas from older women (Zhang *et al.*, 2022). These results demonstrated that maternal age affects the function of specific placental cell types in an apparently normal pregnancy. These effects could be similar, but to a lesser extent, in the placentas involved in adverse pregnancy outcomes owing to maternal aging.

#### Pre-eclampsia

Most studies of placental pathologies have focused on pre-eclampsia (defined as hypertension after 20 weeks of gestation only (Zhou *et al.*, 2022) or associated with high proteinuria as well (Tsang *et al.*, 2017; Zhang *et al.*, 2021a)) that resulted in preterm C-section deliveries. Tsang *et al.* (2017) showed that there is an up-regulation of genes involved in cell death in EVTs in placentas from pre-eclamptic compared to normal pregnancies, implying that EVTs are more affected than other trophoblast types. Moreover, the authors also correlated EVT dysfunction with an increased release of cell-free RNA by the EVTs in maternal blood, providing a potential non-invasive approach for early detection of pre-eclampsia. Through the integrated analysis of Tsang *et al.* and Pique-Regi *et al.* data, one study showed that EVTs were overrepresented while stromal cell types were less abundant (Campbell *et al.*, 2023) and overexpression of *FLT1*, *LEP*, and *ENG* in bulk RNAseq analysis of pre-eclamptic placentas was mainly linked to differences in cell composition (Campbell *et al.*, 2023). On the other hand, Zhang *et al.* identified up-regulation of endoplasmic reticulum-related genes in STBs of pre-eclampsia placentas (Zhang *et al.*, 2021a). Moreover, the expression of genes involved in immune function was enriched in both in vCTBs and EVTs from pre-eclamptic placentas, suggesting altered placental immune function. Interestingly, Zhou *et al.* showed alterations in gene profiles related to immunity, similar to Zhang *et al.* (2021a) and Tsang *et al.* (2017), apoptosis in EVT was also altered in pre-eclamptic samples: *CEBPB* and *GTF2B*, regulating apoptosis and cell invasion respectively, were novel transcription factors identified with reduced activity in pre-eclamptic samples (Zhou *et al.*, 2022). In addition to this, they also observed an enrichment in hormone secretion function in EVTs (Zhou *et al.*, 2022).

#### Gestational diabetes mellitus

The comparison of two placentas from healthy women and two from women with GDM after cesarean delivery highlighted 136 genes up-regulated in GDM trophoblast cells related to estrogen signaling as well as antigen processing and presentation, while the down-regulated genes were mostly related to the inflammatory IL-17 pathway (Yang *et al.*, 2021). In granulocytes and myelocytes, genes involved in the inflammatory response were up-regulated and those involved in actin binding were down-regulated. Of particular interest, an increase in natural killer cells and anti-inflammatory polarized macrophages was observed in GDM placentas compared to controls. Altogether, these results suggest that the most important transcriptomic changes in GDM are related to immune system regulation in the placental microenvironment (Yang *et al.*, 2021).

#### Infectious diseases

Four studies applied scRNAseq to analyze infectious diseases. The first study analyzed placentas from non-infected women at three different GAs with both scRNAseq and snRNAseq (Pique-Regi *et al.*, 2020). They demonstrated that *ACE2* and *TMPRSS2* (canonical cell entry mediators for SARS-CoV-2) had a negligible co-expression in the different placental cells, indicating that this is unlikely to be the entrance pathway of SARS-CoV2 in case of vertical transmission. Further investigations by the same group and others confirmed that SARS-CoV2 infection during pregnancy mainly induced unique inflammatory responses at the maternal–fetal interface (Lu-Culligan *et al.*, 2021; Chen *et al.*, 2022; Garcia-Flores *et al.*, 2022) and that SARS-CoV2 was not detected in placental tissues (Garcia-Flores *et al.*, 2022). However, receptors for Zika and cytomegalovirus, both responsible for congenital malformations, were shown to be expressed by decidual and stromal cells of the placenta, validating their effects at the feto-maternal interface (Pique-Regi *et al.*, 2020).

### Limitation of the meta-analyses

One limitation of both meta-analyses is the number of studies that were included as most studies have not deposited annotated or raw datasets. However, the analytical pipeline provided here can be easily applied in future when more datasets will be available. To help with the annotation of most placental cell types, we provide a list of specific markers, which can be used in further annotation of scRNAseq studies. However, it remains to be determined if these markers will work equally well for snRNAseq data. Furthermore, integrated analysis yielded several hundred DEGs for each cell type. Genes were further selected if they overlapped with our annotated datasets and/or the curated markers. Therefore, more genes could probably be identified as specific markers. Particularly, the validation of markers for fusing-CTB in the literature was challenging as this cell type is transitory. Here, we referred to published STB markers to validate their use as markers and more work is expected to confirm their utility.

### Limitations and perspectives of single-cell RNAseq use in placenta research

Overall, usage of scRNAseq for the analysis of placental transcriptomes in various pathologies has led to new insights and understanding of placental diseases. Furthermore, the use of single-cell analysis in trophoblast organoids has yielded novel information regarding trophoblast differentiation (Shannon *et al.*, 2022; Arutyunyan *et al.*, 2023).

As demonstrated in this review, however, the characterization of cell types in the human placenta is not straightforward and has not been performed optimally in some published scRNAseq studies, which can limit the impact of the results. Indeed, mature STBs were likely missed using scRNAseq and precursors of STBs at the initial step of the cell fusion (fusing-CTB) were captured, limiting the annotation of this placental-specific cell type. Furthermore, cell types from the inner core of the villi, which are particularly rich in stromal cells, that may have important implications for disease pathogenesis were understudied.

Owing to the high cost of scRNAseq studies, most investigations of placental gene expression rely on a limited number of individuals (typically three/group), which poses challenges for robust statistical analysis. This limitation is compounded by the difficulty of obtaining fresh, high-quality cells for analysis. As a result, studies are often unable to consider covariables for more detailed analysis, which could provide valuable insights into the identification of cell types and their potential association with adverse pregnancy outcomes.

To overcome these issues, snRNAseq can be applied, as well as the recently introduced scRNAseq analysis of formalin-fixed paraffin-embedded samples by 10× Genomics, both simplifying the experimental planning and logistics. Unlike scRNAseq, snRNAseq can be performed on frozen tissue, which vastly simplifies experimental planning and offers the additional advantage of increasing recovery of STBs and decidual GCs. In addition, placental RNA integrity is rapidly and easily affected by sampling and storage methods (Fajardy *et al.*, 2009; Huang *et al.*, 2013; Reiman *et al.*, 2017). As high-quality input of single cells (or nuclei) is a key variable for successful scRNAseq analyses, correct handling and sampling is crucial. Notably, in a recent study analyzing post-mortem brain snRNAseq, RNA integrity was only weakly correlated with the final number of recovered cells, and had little effect on the number of genes or UMI detected (Maitra *et al.*, 2021), suggesting that for samples with suboptimal quality and RNA integrity, the main consequence may be simply that more cells fail quality control, leading to less cells recovered overall rather than the preferential loss of specific cell types. Importantly, this assumes that the RNA degradation is evenly distributed across all cell types.

## Conclusions

In this review, we have highlighted how scRNAseq has enabled the identification of previously unknown cell subtypes in the human placenta and provided insight into the molecular mechanisms underlying the differentiation of placental cell types. Despite these advances, there is still a lack of consensus regarding cell type annotations and the genes used to identify them. This also suggests the fallibility of the clustering algorithms for developing tissues when comparing to fully developed tissues, as cells do not have a fixed but a continuous signature throughout development. To assist future studies, here we have outlined specific marker genes using different meta-analyses and the available literature for both first trimester and term placentas (Fig. 7). While application of the single-cell technique to study placentas has traditionally been affected by sampling and lack of consideration for potential confounding factors, such as fetal sex, the use of scRNAseq, snRNAseq, or spatial transcriptomics holds strong promise for enhancing our understanding of human placentas and pregnancy-related disorders in future studies.

## Supplementary Material

dmae006_Supplementary_Data

## Data Availability

The datasets were derived from sources in the public domain [Gene Expression Omnibus (GEO): GSE89497, GSE174481, GSE173193; BioProject ID: PRJNA492324; ArrayExpress: E-MTAB-6701; dbGaP: phs001886.v1.p1]. The data underlying this article are available in the article and in its online supplementary material.
