# Supplementary material for: Revealing the molecular landscape of human placenta: a systematic review and meta-analysis of single-cell RNA sequencing studies
Source: Hum Reprod Update. 2024 Mar 13;30(4):410–41. doi: 10.1093/humupd/dmae006 (PMC11215163; doi:10.1093/humupd/dmae006)
Supplement: dmae006_Supplementary_Data [file dmae006_supplementary_data.zip › Supplementary Figure S2 final.pdf]

**Supplementary Figure S2:** Extended data for first trimester integration.

a) Alignment score matrix of the 4 studies that produce available single-cell RNAseq raw data in first trimester placentas. b) UMAP of the integration data colored by study. c) Heatmap of the top 10 DEGs between trophoblasts vs non-trophoblasts. d) Heatmap of the top 10 DEGs of each placental cell types  single-cell RNAseq data from first trimester placentas

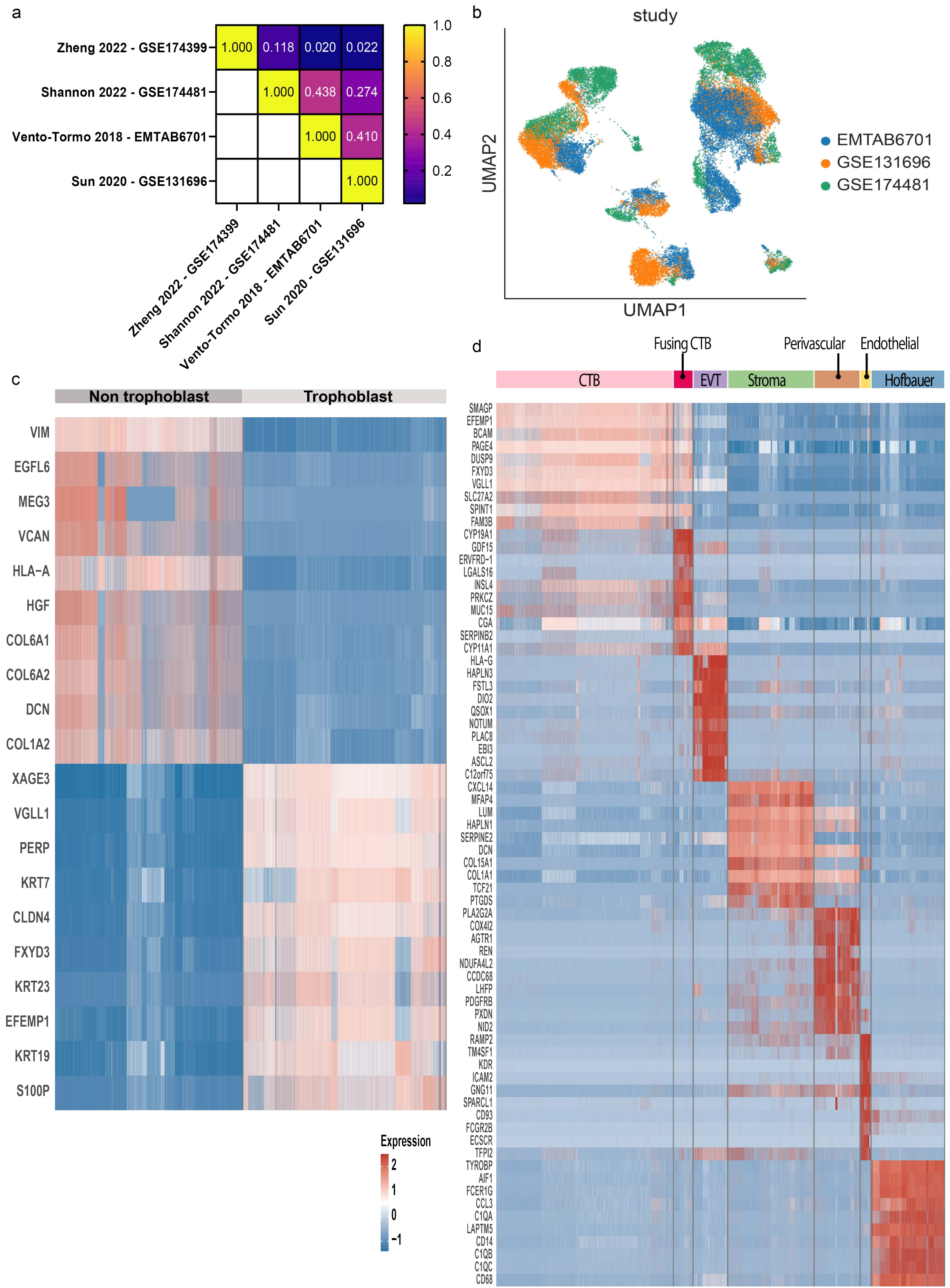

a
Zheng 2022 - GSE174399
Shannon 2022 - GSE174481
Vento-Tormo 2018 - EMTAB6701
Sun 2020 - GSE131696
1.000
0.118
0.020
0.022
1.000
0.438
0.274
1.000
0.410
1.000
1.0
0.8
0.6
0.4
0.2
b
study
UMAP2
UMAP1
EMTAB6701
GSE131696
GSE174481
c
Non trophoblast
Trophoblast
VIM
EGFL6
MEG3
VCAN
HLA−A
HGF
COL6A1
COL6A2
DCN
COL1A2
XAGE3
VGLL1
PERP
KRT7
CLDN4
FXYD3
KRT23
EFEMP1
KRT19
S100P
Expression
2
1
0
−1
d
Fusing CTB
Perivascular
Endothelial
CTB
EVT
Stroma
Hofbauer
SMAGP
EFEMP1
BCAM
PAGE4
DUSP9
FXYD3
VGLL1
SLC27A2
SPINT1
FAM3B
CYP19A1
GDF15
ERVFRD-1
LGALS16
INSL4
PRKCZ
MUC15
CGA
SERPINB2
CYP11A1
HLA-G
HAPLN3
FSTL3
DIO2
QSOX1
NOTUM
PLAC8
EBI3
ASCL2
C12orf75
CXCL14
MFAP4
LUM
HAPLN1
SERPINE2
DCN
COL15A1
COL1A1
TCF21
PTGDS
PLA2G2A
COX4I2
AGTR1
REN
NDUFA4L2
CCDC68
LHFP
PDGFRB
PXDN
NID2
RAMP2
TM4SF1
KDR
ICAM2
GNG11
SPARCL1
CD93
FCGR2B
ECSCR
TFPI2
TYROBP
AIF1
FCER1G
CCL3
C1QA
LAPTM5
CD14
C1QB
C1QC
CD68
