# Supplementary material for: Revealing the molecular landscape of human placenta: a systematic review and meta-analysis of single-cell RNA sequencing studies
Source: Hum Reprod Update. 2024 Mar 13;30(4):410–41. doi: 10.1093/humupd/dmae006 (PMC11215163; doi:10.1093/humupd/dmae006)
Supplement: dmae006_Supplementary_Data [file dmae006_supplementary_data.zip › Supplementary Figure S3 final.pdf]

Supplementary Figure S3: Extended data for term integration.

a) Alignment score matrix of the 4 studies that produce available single-cell RNAseq raw data in term placentas. b) UMAP of the integration data colored by study. c) Heatmap of the top 10 DEGs between trophoblasts vs non-trophoblasts. d) Heatmap of the top 10 DEGs of each placental cell types in single-cell RNAseq data from term placentas

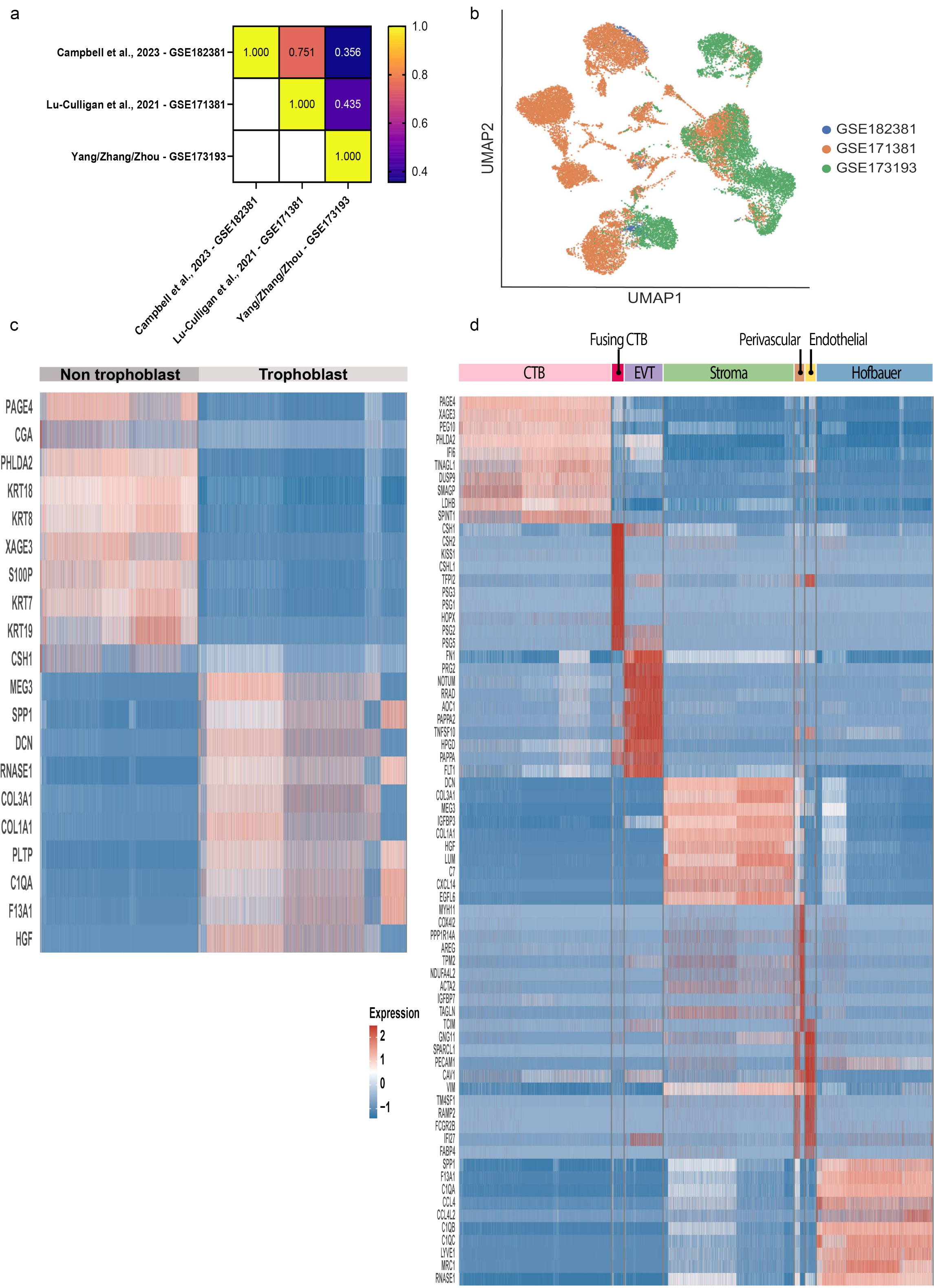
a
Campbell et al., 2023 - GSE182381
Lu-Culligan et al., 2021 - GSE171381
Yang/Zhang/Zhou - GSE173193
1.000
0.751
0.356
1.000
0.435
1.000
1.0
0.8
0.6
0.4
Campbell et al., 2023 - GSE182381
Lu-Culligan et al., 2021 - GSE171381
Yang/Zhang/Zhou - GSE173193
b
UMAP2
UMAP1
GSE182381
GSE171381
GSE173193
c
Non trophoblast
Trophoblast
PAGE4
CGA
PHLDA2
KRT18
KRT8
XAGE3
S100P
KRT7
KRT19
CSH1
MEG3
SPP1
DCN
RNASE1
COL3A1
COL1A1
PLTP
C1QA
F13A1
HGF
Expression
2
1
0
−1
d
Fusing CTB
Perivascular
Endothelial
CTB
EVT
Stroma
Hofbauer
PAGE4
XAGE3
PEG10
PHLDA2
IFI6
TINAGL1
DUSP9
SMAGP
LDHB
SPINT1
CSH1
CSH2
KISS1
CSHL1
TFPI2
PSG3
PSG1
HOPX
PSG2
PSG5
FN1
PRG2
NOTUM
RRAD
AOC1
PAPPA2
TNFSF10
HPGD
PAPPA
FLT1
DCN
COL3A1
MEG3
IGFBP3
COL1A1
HGF
LUM
C7
CXCL14
EGFL6
MYH11
COX4I2
PPP1R14A
AREG
TPM2
NDUFA4L2
ACTA2
IGFBP7
TAGLN
TCIM
GNG11
SPARCL1
PECAM1
CAV1
VIM
TM4SF1
RAMP2
FCGR2B
IFI27
FABP4
SPP1
F13A1
C1QA
CCL4
CCL4L2
C1QB
C1QC
LYVE1
MRC1
RNASE1
