# Supplementary material for: Revealing the molecular landscape of human placenta: a systematic review and meta-analysis of single-cell RNA sequencing studies
Source: Hum Reprod Update. 2024 Mar 13;30(4):410–41. doi: 10.1093/humupd/dmae006 (PMC11215163; doi:10.1093/humupd/dmae006)
Supplement: dmae006_Supplementary_Data [file dmae006_supplementary_data.zip › Supplementary Data File S1 final.docx]

# Supplementary Data File S1 Detailed methods for the meta-analysis.

# Methods

### Meta-analysis flow

An extensive literature search using Pubmed was performed to find articles related to human placental single cell or single nuclei RNA sequencing using the search: “((placenta) AND (single-cell) OR (single-nuclei))” or “((maternal-fetal interface) AND (single-cell) OR (single-nuclei))”. Filters to select full text and Humans related articles were applied. Only articles published before the 31^st^ of March, 2023 were included in this systematic review and meta-analysis (Figure S1). From these results, first, we removed duplicates and excluded articles from their titles and abstracts. Secondly, the eligibility of the articles that were not excluded at step 1, was assessed by reading the full text versions. Selected articles produced new original single cell transcriptomic data from human placental samples. Articles focusing on non-human tissues, on maternal origin cells or only on cultured cells, as well as studies focusing on tumor or embryo cells or that did not perform single cell transcriptomic experiments were excluded. Similarly, articles that used single cell transcriptomic data generated in another study were not considered. Furthermore, we excluded analysis of placental cells from maternal blood or cervix. Studies without access to the full text articles were also excluded.

Furthermore, to capture other potentially relevant articles, we also perform an analysis of already published reviews in this field (Supplementary Fig. S1). Furthermore, when analyzing data deposits, if a new article was identified, it was included in the meta-analysis list (Supplementary Table SX) (**AUTHOR:** are you referring to Supplementary Table S10 here, or another Supplementary Table?).
