# Supplementary figures and images for: Revealing the molecular landscape of human placenta: a systematic review and meta-analysis of single-cell RNA sequencing studies
Source: Hum Reprod Update. 2024 Mar 13;30(4):410–41. doi: 10.1093/humupd/dmae006 (PMC11215163; doi:10.1093/humupd/dmae006)

Supplementary Figure S1: Meta-analysis Flow chart.

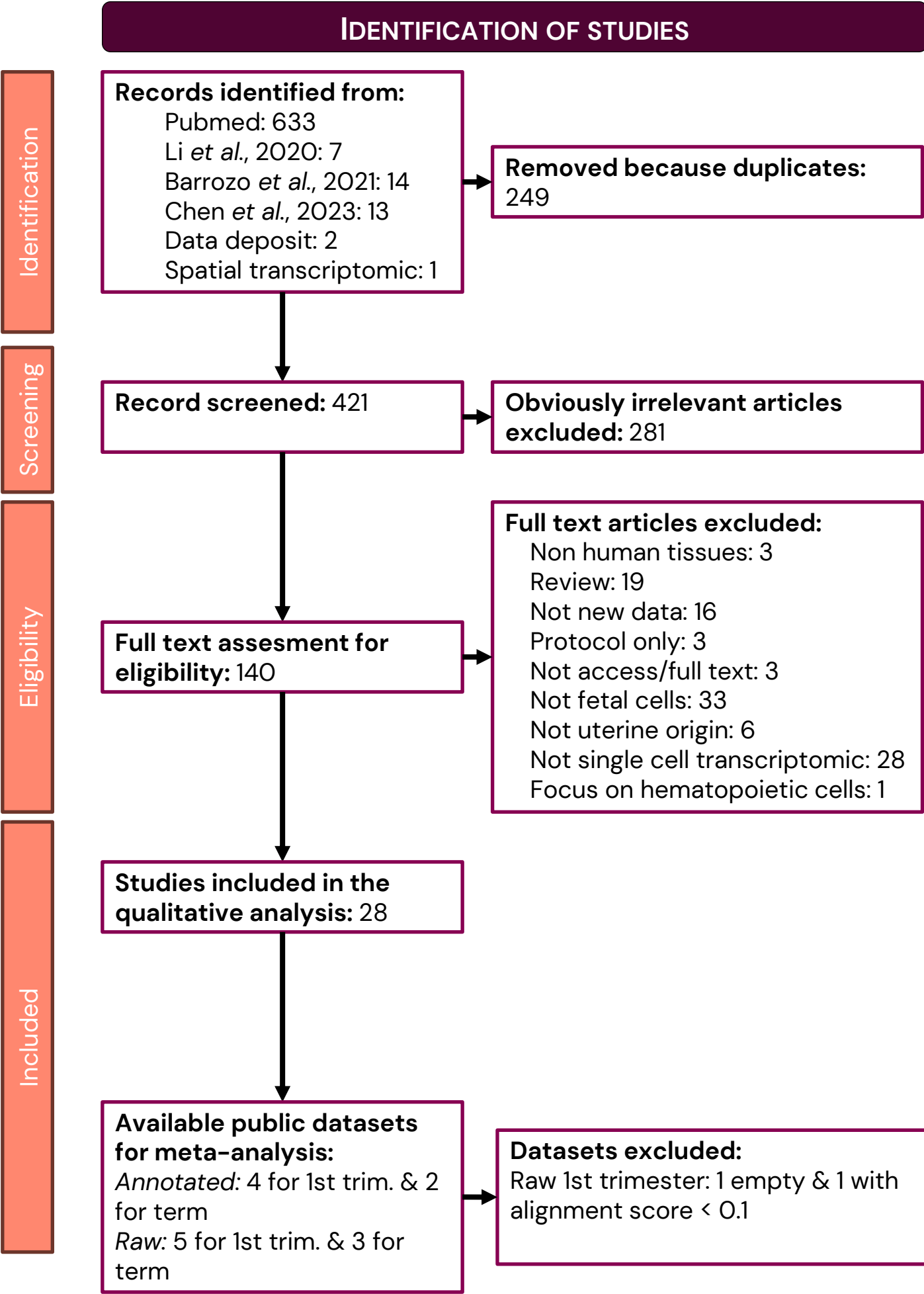

Supplement: dmae006_Supplementary_Data [file dmae006_supplementary_data.zip › Supplementary Figure S1 final.pdf]
